# Degradation of Synthetic Restoration Materials by Xerotolerant/Xerophilic Fungi Contaminating Canvas Paintings

**DOI:** 10.3390/jof11080568

**Published:** 2025-07-30

**Authors:** Amela Kujović, Katja Kavkler, Michel Alexander Wilson-Hernandez, Miloš Vittori, Luen Zidar, Cene Gostinčar, Kristina Sepčić, Yordanis Pérez-Llano, Ramón Alberto Batista-García, Nina Gunde-Cimerman, Polona Zalar

**Affiliations:** 1 Department of Biology, Biotechnical Faculty, University of Ljubljana, Jamnikarjeva 101, SI-1000 Ljubljana, Slovenia; amela_kujovic@hotmail.com (A.K.); milos.vittori@bf.uni-lj.si (M.V.); luen.zidar@nib.si (L.Z.); cene.gostincar@bf.uni-lj.si (C.G.); kristina.sepcic@bf.uni-lj.si (K.S.); nina.gunde-cimerman@bf.uni-lj.si (N.G.-C.); 2 Institute for the Protection of Cultural Heritage of Slovenia, Poljanska 40, SI-1000 Ljubljana, Slovenia; katja.kavkler@zvkds.si; 3 Centro de Investigación en Dinámica Celular, Instituto de Investigación en Ciencias Básicas y Aplicadas, Universidad Autónoma del Estado de Morelos, Cuernavaca CP 62209, Morelos, Mexico; michel.wilson@ibt.unam.mx (M.A.W.-H.); yordanis.perezllano@yahoo.com (Y.P.-L.); rabg@uaem.mx (R.A.B.-G.); 4 Departamento de Biología Animal, Biología Vegetal y Ecología, Universidad de Jaén, 23071 Jaén, Spain

**Keywords:** low water activity, biodeterioration, enzymes, transcriptomics

## Abstract

Canvas paintings are prone to biodeterioration due to their complex chemical composition, which can support fungal growth even under controlled conditions. This study evaluated the susceptibility of common synthetic restoration materials—Lascaux glues (303 HV, 498 HV), Acrylharz P550, BEVA 371, Laropal A81, and Regalrez 1094—to degradation by fourteen xerotolerant/xerophilic fungal strains. All tested *Aspergillus* and *Penicillium* species extensively colonized, especially artificially aged materials. FTIR-PAS analysis revealed chemical changes in carbonyl and C–H bonds in Laropal A81 and Regalrez 1094 colonized by *Aspergillus* spp. Scanning electron microscopy (SEM) imaging showed thinning of Lascaux glues and deformation of Regalrez 1094. Transcriptomic profiling of *A. puulaauensis* grown on Lascaux 498 HV and Regalrez 1094 identified altered expression of genes coding for esterases and oxidases, enzymes involved in synthetic polymer degradation. Esterase activity assays using 4-nitrophenol-based substrates confirmed significant enzymatic activity correlating with the presence of ester bonds. These findings highlight the vulnerability of synthetic restoration materials, specifically Laropal A81, Regalrez 1094, and Lascaux glues, to extremophilic fungi thriving in environments with low water activity. The results emphasize the urgent need for specific knowledge on fungi and their metabolic pathways to use/develop more durable conservation materials and strategies to protect cultural heritage objects from biodeterioration.

## 1. Introduction

Canvas paintings hold an important place within our cultural heritage. They serve as visual narratives of history, culture, and art, capturing moments and expressions across centuries. However, these artifacts are often threatened by microbial growth and microbially induced deterioration. Fungal colonization is particularly problematic, supported by the organic materials present in the paintings or introduced as dirt or dust, and in some cases, occurring even under carefully managed environmental conditions [1]. Typically, microbial growth requires moderate temperatures and high relative humidity (RH); however, xerotolerant and xerophilic fungi can grow at low RH, causing deterioration of canvas paintings stored in buildings with controlled climatic conditions, such as museums, which maintain a temperature range of 16–25 °C and 40–60% RH with a maximum of 5–10% variation over 24 h [2]. Such fungal growth can lead to aesthetic change, but also to mechanical and biochemical degradation, significantly reducing the value and integrity of the affected artworks.

The impact of different synthetic materials used in painting artworks on fungal growth needs to be better understood. Synthetic materials are numerous and diverse, and can appear in various parts of canvas painting: as canvas support (acrylic, nylon, and polyester), paints, and as protective coats [3,4]. Synthetic paints are composed of a pigment (usually mineral), a binder (vinyl acetate, vinyl chloride, acrylate, styrene latex, etc.), and a solvent (hydrocarbon or aqueous) [3]. Contemporary techniques, such as gesso, use an acrylic binder, synthetic paint binders (acrylic, vinyl latex, or polyvinyl acetate (PVA)), and synthetic varnishes.

Synthetic materials are also used in conservation–restoration treatments as a presumably more bioresistant alternative to traditional wax–resin mixtures [5,6]. Among the often used are Acrylharz P550, BEVA 371, Laropal A81, Regalrez 1094, and others. Acrylic resins, such as Acrylharz P550, are composed of acrylates and methacrylates, which are esters of acrylic and methacrylic acids [7]. Mixed resin BEVA 371 consists of numerous components, some of which contain ester bonds [8,9,10,11]. Laropal A81 is a urea–aldehyde resin formed by urea and aliphatic aldehyde [12] condensation; its degradation may occur similarly to the degradation of urea-formaldehyde resin polymers [13]. Regalrez 1094 is a highly stable hydrocarbon resin produced from petroleum-based feedstocks through polymerization and hydrogenation of pure monomer hydrocarbons [14]; its degradation may occur as the breakdown of the long main chains or cleavage of side groups [10]. The long-term outcome of the use of synthetic restoration materials in combination with original ones, coupled with microclimatic conditions, is often difficult to predict and raises questions about their durability and compatibility [3,4,15,16]. Several reports on the biodeterioration of synthetic materials used in cultural heritage conservation–restoration are found in the literature [3,6,17,18,19,20,21], depending on the application methods of synthetic restoration materials, the amount of material applied, and its subsequent aging [5,22]. Growth of fungi on alkyd and acrylic resins, particularly of the genera *Aspergillus*, *Cladosporium*, and *Penicillium*, has been reported; however, the degradation and the exact mechanisms remain poorly understood [6,16,17,18,19,20,21].

Several studies have examined the degradation ability of bacteria [23,24] and fungi [25,26,27,28,29] inhabiting canvas paintings. Microorganisms that colonize synthetic polymers can release metabolites into the material, such as enzymes and pigments, leading to the degradation of the polymer and its additives or residual monomers [30]. Enzymes such as esterases/lipases, proteases, and ureases, which have the ability to cleave ester bonds, play a significant role in this process [31]. Among fungi, the genera *Aspergillus*, *Cladosporium*, and *Penicillium* pose significant threats due to their broad range of enzymatic activities [26,32]. The enzymes these fungi most commonly excrete include cellulases (β-glucosidases, endoglucanases), esterases (including lipases and lecithinases), amylases, ureases, and proteases (caseinase, gelatinase), among others [26,27,29,33,34,35]. Additionally, the microbial ability to break down hydrocarbons is crucial in degrading synthetic materials [36]. In particular, fungi of the genera *Aspergillus* and *Penicillum* can excrete organic acids [26,27,32,37], contributing to irreversible changes in the appearance and structure of the material [21,38]. Fungi commonly found on alkyd and acrylic resins, such as xerotolerant/xerophilic species of *Aspergillus*, *Aureobasidium*, *Cladosporium*, and *Penicillium*, exhibit esterase and urease activities and can assimilate hydrocarbons. However, xerophilic species, known to often attack paintings in environments with controlled conditions, have not yet been studied with sophisticated methods in connection with metabolic activity while growing on synthetic restoration materials, and their impact on the long-term preservation of such paintings remains insufficiently understood [29].

This study aimed to investigate the susceptibility of synthetic materials, commonly used in the conservation and restoration of canvas paintings, to degradation by xerotolerant/xerophilic fungi isolated from paintings in a previous study [29]. Recent findings have indicated that these fungi are frequently found on historical paintings, challenging the assumption that low humidity in cultural heritage facilities prevents fungal growth altogether [39]. Based on our previous study [29], fourteen fungal strains representing fourteen species from eight genera were selected based on their enzymatic profiles and tested for their ability to degrade synthetic materials. The key criteria for selection were the esterase and urease activities, along with their ability to assimilate hydrocarbons. Laboratory samples with non-aged and aged synthetic restoration materials applied onto glass slides were inoculated by fungi, and after incubation, viewed by FTIR-PAS and SEM. The transcriptome of the selected fungus, *Aspergillus puulaauensis*, was accessed while growing on selected material, and differentially expressed genes encoding enzymes were determined.

This study aimed to investigate the susceptibility of synthetic materials commonly used in the conservation and restoration of canvas paintings to degradation by xerotolerant and xerophilic fungi. These fungal strains were previously isolated from historical paintings [29], challenging the prevailing assumption that low humidity conditions in cultural heritage institutions are sufficient to prevent fungal growth [39]. Fourteen fungal strains, each representing a distinct species from eight different genera, were selected for their enzymatic potential to degrade synthetic materials. Selection criteria included esterase and urease activity, as well as the ability to assimilate hydrocarbons. Synthetic restoration materials, both non-aged and artificially aged, were applied to glass slides, inoculated with the selected fungi, and incubated under controlled conditions. Fungal colonization and material degradation were then analyzed using Fourier-transform infrared photoacoustic spectroscopy (FTIR-PAS) and scanning electron microscopy (SEM). In addition, transcriptomic analysis of *Aspergillus puulaauensis* grown on selected synthetic materials was performed to identify differentially expressed genes encoding enzymes involved in polymer degradation.

## 2. Materials and Methods

### 2.1. Film Preparation and Artificial Aging

Fungal growth was tested on selected restoration materials (listed in Table 1) as the sole nutrient source applied to glass slides. Skin glue was used as a positive control and prepared as a ten percent (*w*/*v*) solution in distilled water; a solution of Laropal A81 was prepared from 20 g of Laropal A81 and 40 g of Shellsol A (Kremer Pigmente, Aichstetten, Germany); and a solution of Regalrez 1094 was prepared from 20 g of Regalrez 1094 and 50 g of xylene (Merck, Darmstadt, Germany).

Glass slides were washed with 96% ethanol (Pharmachem, Ljubljana, Slovenia), rinsed with water and dH_2_O, and dried in a drying oven at 110 °C. The materials were then evenly applied as a thin film to the glass slides with a brush. Rabbit skin glue was applied in two layers and was used as a positive control for fungal growth, as proteinaceous materials are the most susceptible to microbial attack on canvas paintings [39]. After drying the coated slides at room temperature (21 °C) for 24 h, half were exposed to UV-A and UV-B light for 300 h using an ARCADIA Fluorescent Lamp High Output T5 D3+ Dragon (14% UV-B; Essex Reptile, Braintree, UK), in accordance with the ISO 11507:2007(E) standard [40]. The remaining slides were stored at room temperature under natural day/night light conditions.

**Table 1:** Materials used in the study and their chemical composition.

Material	Form Used in This Study	Usage	Chemical Composition	Reference
Skin Glue	Solution (Samson Kamnik, Kamnik, Slovenia)	Consolidation, local sanation, gluing	Gelatin and other protein residues of collagen, keratin, or elastin	[1]
Lascaux Acrylic Glue 303 HV	Original form (Lascaux Colours & Restauro, Wangen-Brüttisellen, Switzerland)	Consolidation, local sanation, gluing	Thermoplastic copolymeric butyl-methacrylate dispersion, thickened with acrylic ester acid	[41]
Lascaux Acrylic Glue 498 HV	Original form (Lascaux Colours & Restauro, Wangen-Brüttisellen, Switzerland)	Consolidation, local sanation, gluing	Thermoplastic copolymeric butyl-methacrylate dispersion, thickened with acrylic ester acid	[42]
Acrylharz P550	Original form (Lascaux Colours & Restauro, Wangen-Brüttisellen, Switzerland)	Consolidation	Organic solution of an acrylic resin on the basis of butyl methacrylate (diluted in white spirit)	[43]
Laropal A81	Solution (Kremer Pigmente, Aichstetten, Germany)	Varnish, binder for pigments	Aldehyde resin-condensation product of urea and aliphatic aldehydes	[12,44]
BEVA 371	Original form (CTS, Briosco, Italy)	Consolidation, local sanation, gluing	Ethylene vinyl acetate copolymer (Elvax 150), cyclohexanone resin (LaropalK80), ethylene vinyl acetate copolymer (A-C 400), phtalate ester of hydroabietyl acid (Cellolyn 21), and paraffinic hydrocarbons (Paraffin). Solvents make up 60% of the solution and are a combination of toluene and petroleum.	[45]
Regalrez 1094	Solution (Kremer Pigmente, Aichstetten, Germany)	Varnish	Low-molecular-weight hydrocarbon resin, formed by a hydrogenated oligomer of styrene and alpha-methyl styrene. It is produced by polymerization and hydrogenation of pure monomeric hydrocarbons.	[14,44]

### 2.2. Fungal Strains, Inocula Preparation, and Culture Conditions

The fungal strains were obtained from deteriorated paintings on canvas exposed in sacral buildings or authorized restoration depository (Table S1), selected from our previous studies [36,41]. The strains were tested for their ability to degrade synthetic materials and selected based on strong enzymatic and other activities [29], and their frequency of isolation from canvas paintings [39]. The strains are maintained in the Culture Collection of Extremophilic fungi Ex within the Infrastructural Centre Mycosmo, part of MRIC UL (Department of Biology, Biotechnical Faculty, University of Ljubljana, Ljubljana, Slovenia). Fresh cultures were grown on Malt Extract Agar (MEA; Biolife Italiana, Milan, Italy; 0.02% malt extract, 0.02% glucose, 0.001% peptone, 0.02% (*m*/*v*) agar; pH = 5–5.5) [46] or Dichloran Glycerol Agar (DG18; Biolife Italiana, Milan, Italy), prepared according to the manufacturer’s instructions [47]. Spore suspensions were prepared in a concentration of 10^7^ spores/mL in 0.9% NaCl for xerotolerant fungi and in 10% NaCl for obligate xerophiles.

Glass slides with applied films of synthetic materials were UV-irradiated for 15 min at 254 nm, on the side of material application. In contrast, the other side of the slide was wiped with 70% ethanol. Glass slides were three-point inoculated with 25 µL of fungal inoculum per point, with added 0.2% glucose for growth initiation. Application of 0.9% NaCl or 10% NaCl solution to a cleaned glass slide was used as a negative control.

Obligate xerophilic strains were incubated in plastic containers (37 × 26 × 14 cm) for 2 months at 24 °C, in an atmosphere with 70% RH. Due to their slow growth, after 2 months of incubation, the RH was increased to 90% and the fungi were further incubated under these conditions for 5 months. The rest of the fungi were incubated for 2 months at 24 °C at a constant relative humidity of 90% RH. To obtain 90% RH, 300 mL of sterile dH_2_O was placed in a box, and for 70% RH, 200 mL of saturated NaCl solution (72 g/200 mL) per box was used. The RH inside the box was measured daily with an RH meter (Conrad Electronic, Ljubljana, Slovenia) placed inside the box throughout the incubation period [48,49].

### 2.3. Microscopic Analysis of Fungal Growth

Fungal growth was observed on glass slides under a Dino-Lite Edge digital microscope (AM7515MT8A; Dino-Lite, Almere, The Netherlands). Digital images were acquired with RS image, version 1.2.3. Growth was scored according to the following criteria: (−) no growth; (+) weak growth with mycelium covering less than half of the inoculated area; (++) moderate growth, characterized by mycelium covering approximately half of the inoculated area (diameter 5 mm); and (+++) strong growth, where mycelium completely outgrew the inoculated spot and even spread over it to glass surface.

Changes in the microstructure of materials overgrown by the most proliferating fungus, *A. puulaauensis*, were examined by scanning electron microscopy (SEM) JSM-7500F, JEOL, Tokyo, Japan. Glass slide circles of 9 mm diameter covered with a layer of test material were cut with a glass cutter and inoculated as previously described (Section 2.2). The fungus was grown in plastic containers (37 × 26 × 14 cm) for 2 months at 24 °C and 90% RH. Samples were fixed with glutaraldehyde vapor, air-dried, and sputter-coated with platinum using an SCD 050 sputter coater, Bal-Tec, Balzers, Liechtenstein. The glass circles were carefully broken in half, resulting in fine cross-sections of growing colonies. Images were obtained using a JSM-7500F field emission scanning electron microscope (JEOL).

### 2.4. FTIR-PAS Analysis

The glass slides with visible growth on films of test materials were cut into 9 mm diameter circles using a manual glass cutter. Infrared (IR) spectra of the test materials were recorded on a Spectrum GX Fourier-transform IR spectrometer (PerkinElmer, Bülach, Switzerland) equipped with a Model 300 photoacoustic detector (MTEC Photoacoustics, Ames, IA, USA). Each sample was scanned in triplicate between 4500 cm^−1^ and 450 cm^−1^ with a resolution of 8 cm^−1^. The spectra obtained are the average of 64 scans. Structural changes were observed after comparison between the control and moderately or severely overgrown resins, where at least 50% of the 9 mm diameter circles were overgrown.

### 2.5. Extracellular Esterase Activity Assay

#### 2.5.1. Cultivation Conditions

Cultures were grown in a medium of 0.17% (*w*/*v*) yeast nitrogen base (YNB; Becton and Dickinson, Franklin Lakes, NJ, USA), 0.5% (NH_4_)_2_SO_4_, 0.1% glucose, and pH = 7. Separately, 10% Tween-80 (*w*/*v*) was prepared in dH_2_O, autoclaved, and aseptically added to the liquid media to a final concentration of 0.1% (*w*/*v*). For the cultivation of obligate xerophiles, the water activity of the liquid medium was lowered by adding 10% NaCl (*w*/*v*). The medium was inoculated with 20 µL of the conidia/spore suspension. After one week of incubation on a rotary shaker (180 rpm) at 24 °C, the cells were centrifuged for 5 min at 5000 rpm (Centrifuge 5810 R, Eppendorf, Leipzig, Germany). The supernatant was collected and immediately frozen at −80 °C. The concentration of total proteins in the supernatant was determined using the BCA Protein Assay Kit (Thermo Scientific, Waltham, MA, USA).

#### 2.5.2. Preparation of Substrates and Measurement of Activity Using UV-VIS Spectrophotometry

Esterase activity was determined against 4-nitrophenyl (4-NP) esters with different acyl chain lengths as substrates in the standard assay [50,51]: 4-nitrophenyl acetate (4NPC2), 4-nitrophenyl butyrate (4NPC4), 4-nitrophenyl octanoate (4NPC8), and 4-nitrophenyl palmitate (4NPC16) (all Sigma-Aldrich, Steinheim, Germany). The activity was measured using a substrate mixture consisting of 50 mM of 4-NP ester in methanol (4NPC2), acetonitrile (4NPC4, 4NPC8), or ethanol (4NPC16, with 80 min of sonication at 37 °C). The substrate mixture was further dissolved in phosphate buffer [100 mM Na_3_PO_4_, 150 mM NaCl, 0.5% (*v*/*v*) Triton X-100, pH = 7.2] at a ratio of 1:9 (*v*/*v*). The reaction was initiated by adding 10 µL of ten-fold-diluted substrate solution to 100 µL of fungal supernatant and carried out for 10 min at 37 °C. The final concentration of the substrate in the reaction mixture was 0.45 mM. Enzyme activity was determined spectrophotometrically at 405 nm using a TECAN Infinite F Nano Plus microplate reader (Tecan Trading AG, Männedorf, Switzerland), after stopping the reaction by cooling the sample on ice. The concentration of 4-NP substrate released was determined from a standard curve obtained for a set of standards of 4-nitrophenyl phosphate (0–500 nmol; Sigma-Aldrich, St. Louis, MO, USA). One unit of esterase activity was defined as the amount of enzyme that released one µmol of 4-nitrophenol per minute.

#### 2.5.3. Statistical Analyses

Two-way ANOVA followed by Tukey’s multiple comparisons test was performed using GraphPad Prism version 10.0.0 for Windows (GraphPad Software, Boston, MA, USA, www.graphpad.com) (*p* = 0.05).

### 2.6. Transcriptome Sequencing of Aspergillus puulaauensis (EXF-7678) Exposed to Lascaux 498 HV and Regalrez 1094

Whole transcriptomes were sequenced for *A. puulaauensis* (EXF-7678) exposed to two materials that caused the most changes, namely, the acrylic dispersion Lascaux 498 HV and the hydrocarbon resin Regalrez 1094.

#### 2.6.1. Cultivation of the Fungus

Biomass was grown in liquid YNB medium (1.7 g/L) with (NH_4_)_2_SO_4_ (5 g/L) supplemented with 0.2% glucose (*w*/*v*) for initial growth, with a final pH = 7. The biomass (1 g/50 mL) was then transferred using a cell strainer (Thermo Scientific, Waltham, MA, USA) to fresh liquid YNB medium, supplemented with ammonium sulfate only (control medium). Fungal enzyme expression was assessed in the same medium supplemented with 3% (*w*/*v*) Lascaux 498 HV and Regalrez 1094, which served as the sole alternative sources of carbon. The cells were kept in this medium for 7 days at 24 °C, and, after that, centrifuged for 5 min at 5000 rpm using the centrifuge 5810 R (Eppendorf, Germany). The harvested biomass was stored at −80 °C until the total RNA was isolated.

#### 2.6.2. RNA Isolation and Transcriptome Sequencing

Total RNA was isolated using a modified TRIzol extraction method (Thermo Fisher, Waltham, MA, USA) [52]. An amount of 100 mg of biomass was homogenized with a mortar and a pestle using liquid nitrogen and transferred to a 1.5 mL RNase-free microcentrifuge tube. An amount of 1 mL of TRIzol reagent (Thermo Fisher, USA) was added to the biomass, followed by a 5 min incubation at room temperature (22 °C). After that, 200 µL of chloroform (Merck, Darmstadt, Germany) was added to the microcentrifuge tube, shaken gently, and centrifuged for 5 min at 10,000 rpm (Eppendorf Centrifuge 5424 R). Then, 450 µL of the supernatant was added to 1350 µL of 100% cold ethanol (on ice). The microcentrifuge tube was inverted five times and left for 5 min on ice, and centrifuged again for 5 min at 10,000 rpm. The supernatant was poured off, and 1 mL of 70–75% ethanol was added, inverted five times, and left on ice for 5 min. After 5 min centrifugation at 14,000 rpm, the supernatant was poured off, and the microcentrifuge tube was turned over so the remaining liquid evaporated (20 min at room temperature). The dried pellet was dissolved in 20 µL of RNase-free water (Qiagen, Hilden, Germany) and stored at −80 °C.

The RNA concentration was measured on a NanoDrop 2000 spectrophotometer (Thermo Scientific). Quality control and library preparation of the samples were performed at Novogene (Novogene, Cambridge, UK), where the sequencing also took place, using Novaseq 6000. Transcriptome sequencing was performed in three independent biological replicates per condition.

#### 2.6.3. Transcriptome Assembly and Analysis

The quality of the raw reads was checked using the program Fastp (v0.23.2) [53], which also removed the adapter sequences and low-quality reads. In addition, we checked the possibility of RNA contamination using the Kraken2 database [54,55]. The fungal transcriptome was assembled de novo using Trinity (v2.15.1) [56,57]. Reads were mapped to the de novo assembly using Salmon (v0.14.1) [58]. The clustering of assembled transcripts and extraction of isoforms was performed using the MMseqs2 tool [59,60,61,62]. The completeness of the assembled transcriptome was verified with BUSCO (v5.5.0) [63]. Differential gene expression analysis was performed in the DESeq2 package in the R environment [64]. Functional annotation was made by the Trinotate software [65], and identification of open reading frames or prediction of coding regions was performed by the TransDecoder tool (v5.7.1) [57]. For functional annotation, we used the online database EggNOG [66]. Using the online tool SignalP [67,68], differentially expressed genes were checked to see if their predicted proteins have signal sequences for targeting to the secretory (Sec) pathway. Functional groups of genes that would contain statistically significantly more or less differentially expressed genes than chance were searched for with Fisher’s exact test with control of false positive results (“false discovery rate”) in the R environment (*p* > 0.05). The data were deposited under BioProject PRJNA1145172 in the NCBI BioProject online database [69].

## 3. Results

### 3.1. Microscopic Analysis of Fungal Growth

**Figure 1:**
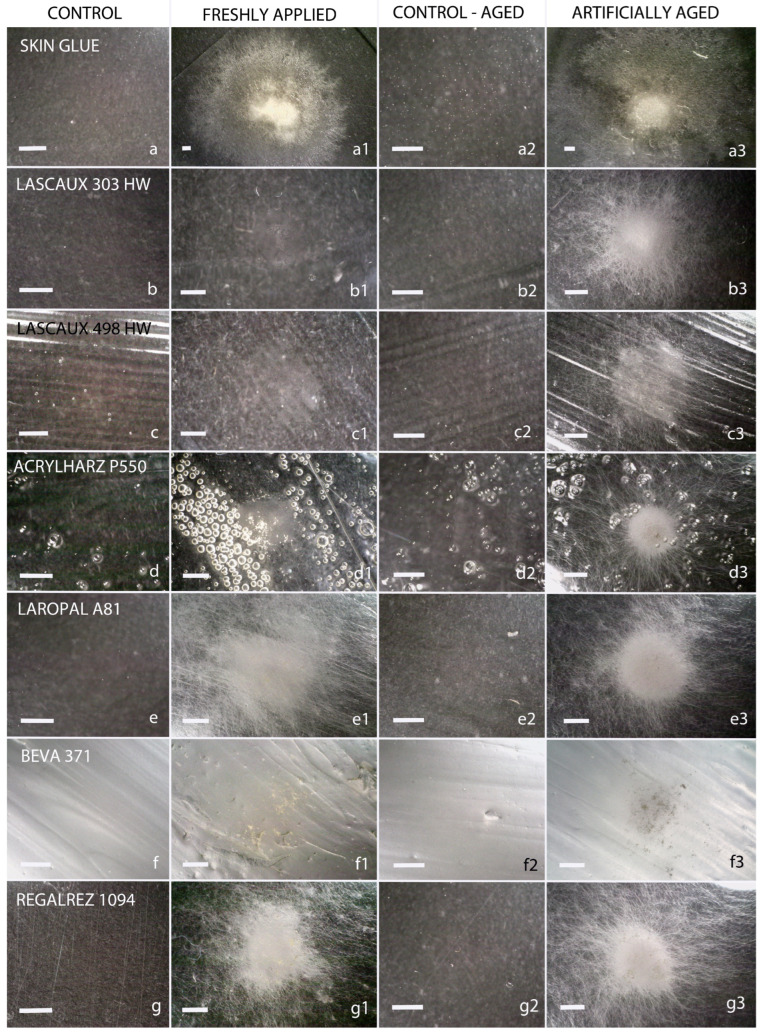
Growth of the facultatively xerophilic *Aspergillus puulaauensis* on non-aged (second column) and artificially aged materials (fourth column), as observed under a USB microscope. The tested materials include: skin glue (**a**–**a3**), Lascaux 303 HV (**b**–**b3**), Lascaux 498 HV (**c**–**c3**), Acrylharz P550 (**d**–**d3**), Laropal A81 (**e**–**e3**), BEVA 371 (**f**–**f3**), and Regalrez 1094 (**g**–**g3**). Scale bars in all images represent 1 mm. The first column shows freshly applied, non-inoculated control samples, while the third column displays artificially aged, non-inoculated controls (from left to right).

The growth of xerophilic fungi on synthetic materials, as assessed by light microscopy, is summarized in Table 2. Representative images of colonization by *Aspergillus puulaauensis*—one of the most extensively growing fungal strains in this study—are shown in Figure 1.

Generally, the most successful fungi in colonizing different materials were species of *Aspergillus* and *Penicillium*, along with *Beauveria pseudobassiana*, which were able to overgrow all tested materials. *Cladosporium cladosporioides* also showed significant growth, overgrowing five materials. Other species were less successful, particularly *Aureobasidium pullulans* and the obligately xerophilic *Wallemia* sp. (aff. *W. muriae*), which did not grow on any of the tested materials.

The most susceptible materials to fungal growth were Laropal A81 and Regalrez 1094, which were overgrown by 10 to 12 species of fungi. These were followed by Acrylharz P550, BEVA 371, Lascaux 498 HV, and Lascaux 303 HV. Acrylic polymers (Lascaux glues and Acrylharz P550) exhibited greater resistance when non-aged and inoculated. Interestingly, all tested materials became equally susceptible to fungal growth after artificial aging, with nine fungi species overgrowing them, except for Laropal A81 and Regalrez 1094.

**Figure 2:**
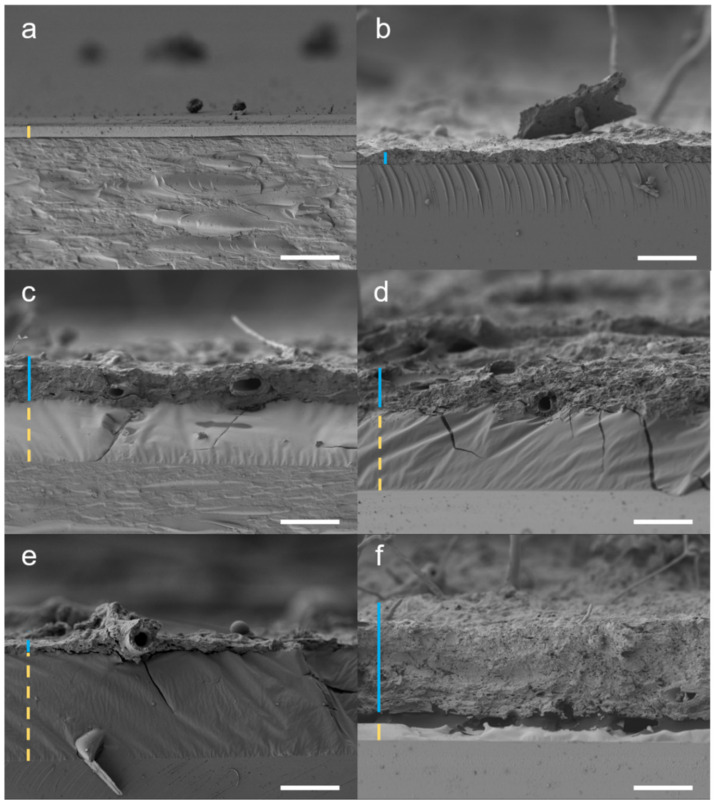
Scanning electron micrographs showing changes in synthetic restoration materials inoculated by *Aspergillus puulaauensis* EXF-7678 after 60 days of incubation at 24 °C at 90% RH: (**a**) uninoculated skin glue (control), (**b**) disappearance of aged skin glue layer, (**c**) thinning of Lascaux 303 HV under hyphae, (**d**) thinning of aged Lascaux 303 HV under hyphae, (**e**) thinning of Lascaux 498 HV under hyphae, and (**f**) deformation of Regalrez 1094 after fungal overgrowth. Layer of the mycelium is marked with blue line. Applied materials are marked with yellow dashed line above the glass slide. All the micrographs were taken under magnification of 2000×. Scale bars = 10 µm.

Microscopic changes in restoration materials were observed using SEM after overgrowth by *Aspergillus puulaauensis* (Figure 2), which also showed the most substantial changes in FTIR-PAS spectra of materials among xerophilic fungi.

The analysis revealed the presence of hyphae, indicating active fungal growth. Layers of non-aged and aged skin glue (positive control) completely disappeared, while other materials showed more resistance to fungal deterioration. Thinning of aged and non-aged Lascaux 303 HV, aged Lascaux 498 HV, and non-aged Regalrez 1094 was observed under hyphae. This phenomenon was not observed in other materials. Additionally, hyphae did not penetrate the materials, as they were not observed within the layers. No noticeable uniform thinning of the materials was observed, except in skin glue, and differences between non-aged and aged materials were not evident as the fungus overgrew all of them.

### 3.2. FTIR-PAS Analysis

Based on the observed fungal growth, glass slides with materials were selected for further analysis of chemical degradation. Of the fungi tested, only some exhibited sufficient growth to be analyzed (Tables S2 and S3). Consequently, analyses were performed on materials overgrown by eight fungal species: all *Aspergillus* and *Penicillium* species and *W. canadensis*. The primary changes were observed in the vibrations of the carbonyl band (1700–1750 cm^−1^) and/or the vibrations of the carbon–hydrogen bond (C-H bond) (2900 cm^−1^). Additional changes were noted in amide bonds (1500–1700 cm^−1^), likely due to fungal growth, and water vibrations (3500–3000 cm^−1^).

**Figure 3:**
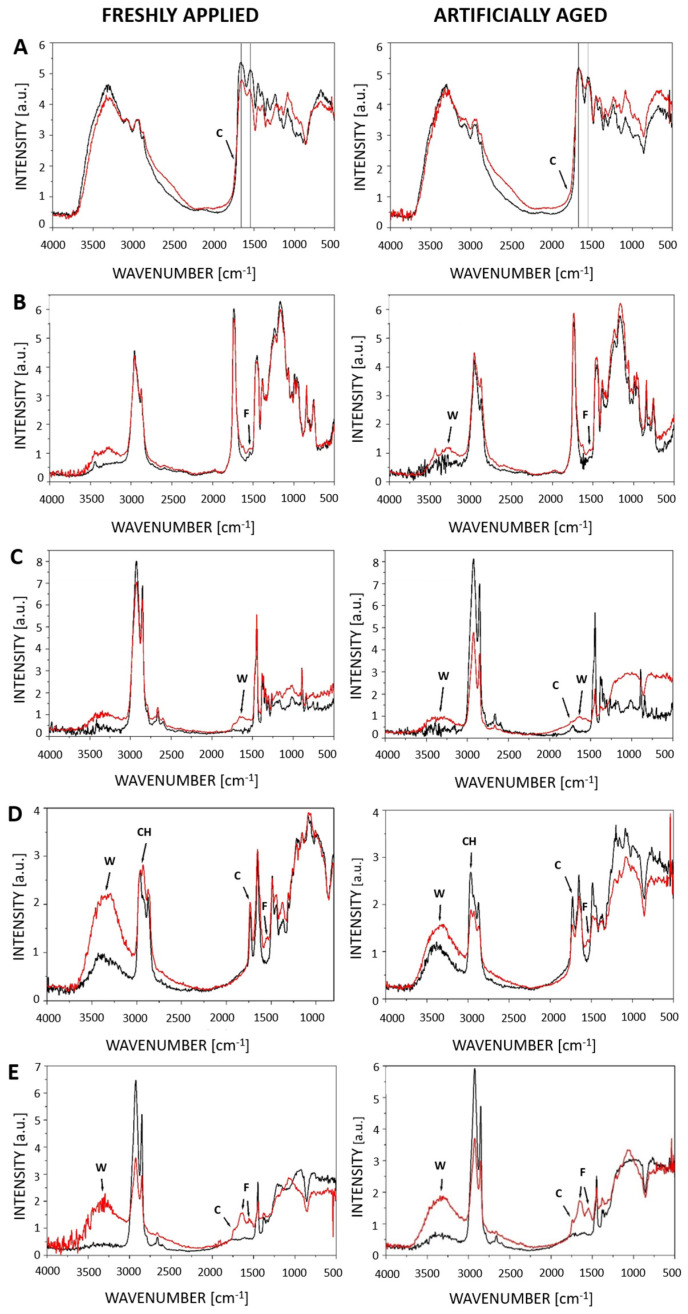
Main changes in the spectra of fresh and aged synthetic materials infected with (**A**–**C**) *Aspergillus puulaauensis* (**A**—skin glue, **B**—Lascaux 498 HV, **C**—Regalrez 1094) and (**D**,**E**) *Aspergillus destruens* (**D**—BEVA 371, **E**—Regalrez 1094), recorded with FT-IR PAS. The spectra of fresh materials are shown on the left, and the spectra of artificially aged ones are on the right. The reference spectra of the non-inoculated materials are shown in black, and the spectra of materials inoculated with the fungus are shown in red. The changes are marked with W—changes in water; C—changes in the carbonyl band region; CH—changes in the C-H bonds; F—changes in the amide bonds.

Among the xerophilic fungi, the most significant chemical alterations were detected in the materials colonized by facultatively xerophilic *A. puulaauensis* (Figure 3). Changes in carbonyl bands were particularly evident in Regalrez 1094 and unaged Lascaux 498 HV, indicating potential degradation of these resins. Furthermore, significant changes in the carbonyl bands were observed in the spectra of *P. chrysogenum* in aged Regalrez 1094 (Table S2). Among obligately xerophilic fungi (Table S3), the most notable changes were caused by *A. destruens*, visible in carbonyl bands of Laropal A81 and Regalrez 1094 (Figure 3). This fungus also induced alterations in the C-H bond vibrations of Regalrez 1094. Similarly, *A. vitricola* also caused changes in the carbonyl bands of Regalrez 1094 and Lascaux 498 HV, as well as alterations in C-H bond vibrations in aged Laropal A81 (Table S3).

Further spectral changes, such as the appearance of amide bands and increased water bands, were particularly notable in materials overgrown by *A. puulaauensis* (Table S3). This fungus caused changes in the amide and water bands in a variety of materials, including Acrylharz P550, BEVA 371, Regalrez 1094, aged Lascaux glues, and unaged Laropal A81. *Penicillium chrysogenum* induced changes in the amide bands of non-aged Lascaux 303 HV (Table S2). These changes were present in the spectra of all materials contaminated by the tested obligate xerophiles, confirming the presence of fungi on the surface of the samples, except for non-aged Lascaux glues (Table S3).

Some fungi could grow on the materials without causing any changes in their chemical structure, including *Aspergillus pseudoglaucus*, *Chaetomium globosum*, *Parengyodontium album*, *Penicillium chrysogenum*, *P. corylophilum*, and *Wallemia canadensis* (Table S2). Likewise, some obligate xerophilic fungi, including *A. destruens* on non-aged Lascaux glues, and *Aspergillus vitricola* on BEVA 371, non-aged Lascaux 498 HV, and aged Acrylharz P550, grew on the materials without causing significant spectral changes (Table S3).

Among the materials deteriorated by xerophilic fungi, Regalrez 1094 exhibited the most significant chemical changes, particularly in the carbonyl bands and/or water or amide bands (Figure 3, Tables S2 and S3). Fewer changes were noted in the spectra of Lascaux glues, primarily as changes in water bands and amide vibrations. Alterations in carbonyl bands were also detected in Lascaux 498 HV degraded by *Aspergillus puulaauensis* (Figure 3, Table S2). Overall, the materials most susceptible to spectral changes induced by obligately xerophilic fungi were Laropal A81 (changes in carbonyl and C-H vibrations), Regalrez 1094, and Lascaux 498 HV (changes in carbonyl bands).

### 3.3. Extracellular Esterase Activity Assay

#### 3.3.1. Spectrophotometric Measurements of Esterase and Lipase Activity

The esterase activity of the tested fungal strains varied significantly depending on the substrate. Figure S1 presents data on the esterase activity of selected fungal strains, with detailed results for each substrate shown in Table S4. The most active on 4-NP acetate were *A. pullulans* (61.5 μmol min^−1^ mg^−1^) and *C. globosum* (38.2 μmol min^−1^ mg^−1^), while the rest of the fungi exhibited lower average specific activity. One of the most preferred substrates for all fungi tested was 4-NP butyrate, with five fungi displaying specific activity above 100 μmol min^−1^ mg^−1^: *Parengyodontium album* (181.4), *Cladosporium cladosporoides* (173.4), *Aureobasidium pullulans* (164.7), *Chaetomium globosum* (121.1), and *Penicillium corylophilum* (99.5). *Beauveria pseudobassiana* had a specific activity around 50 μmol min^−1^ mg^−1^ (47.4), while the other fungi were less successful. Only *Wallemia* species, *Aspergillus destruens*, and *A. vitricola* did not hydrolyse this substrate. The second most preferred substrate was 4-NP octanoate, with *Aureobasidium pullulans* showing specific activity exceeding 100 μmol min^−1^ mg^−1^ (119.6). Six xerophilic fungi demonstrated particular activity above 50 μmol min^−1^ mg^−1^: *Chaetomium globosum* (87.1), *Cladosporium cladosporoides* (85.1), *Parengyodontium album* (70.7), *Aspergillus puulaauensis* (58.8), *Penicillium corylophilum* (56.7), and *Beauveria pseudobassiana* (56.2). The remaining fungi degraded the substrate to a lesser extent. The most successful in degrading 4-NP palmitate were *Cladosporium cladosporoides* (60.4 μmol min^−1^ mg^−1^) and *Chaetomium globosum* (45.4 μmol min^−1^ mg^−1^). Other fungi were less successful, though all could use this substrate.

In general, obligate xerophiles exhibited lower esterase or lipase activity, with specific activities toward all substrates remaining below 20 μmol min^−1^ mg^−1^. The obligately xerophilic aspergilli displayed minimal activity toward most substrates, except 4-NP butyrate, where their activity was nearly absent. One fungus, *Wallemia* sp. (aff. *muriae*), did not show any activity.

#### 3.3.2. Statistical Analysis of Esterase Assay

**Figure 4:**
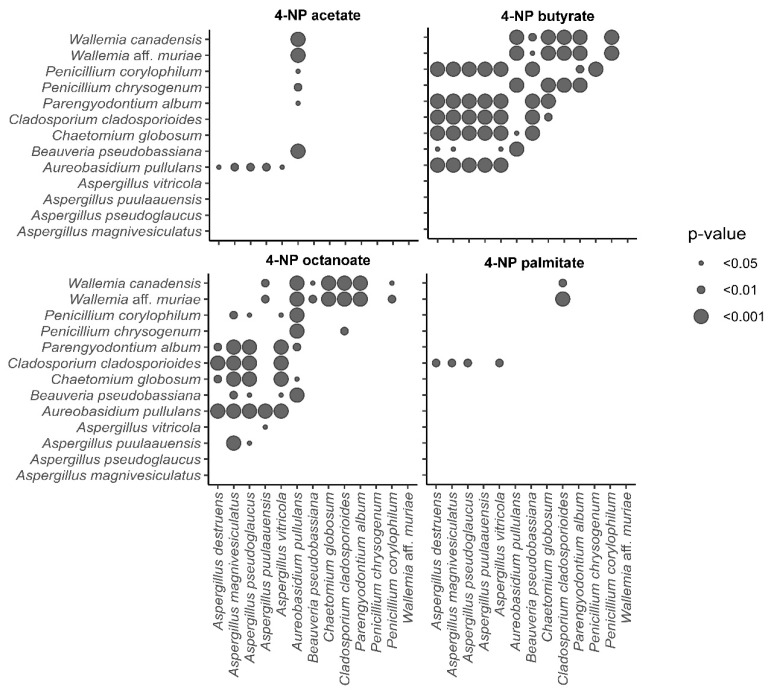
Graphical representation of two-way ANOVA analysis of enzymatic activities for 4-NP acetate, 4-NP butyrate, 4-NP octanoate, 4-NP palmitate. Dots represent significant differences between two species (*p* < 0.001; *p* < 0.01; *p* < 0.05).

The results of the statistical analysis by two-way ANOVA (Figure 4) indicate that the different species were similarly active on 4-NP acetate and 4-NP palmitate. At the same time, there were significant differences between the tested species (*p* < 0.001), particularly in the degradation of 4-NP of butyrate and 4-NP of octanoate, suggesting different abilities of the strains to degrade the latter two and possible specific esterolytic activity. The most utilized substrate was 4-NP butyrate, followed by 4-NP octanoate, 4-NP palmitate, and 4-NP acetate, as shown in Figure S1.

### 3.4. Transcriptome Sequencing of Aspergillus puulaauensis (EXF-7678) Exposed to Lascaux 498 HV and Regalrez 1094

Of the universally present single-copy proteins within fungal genomes, the vast majority (>99%) were present in the de novo assembled transcriptome, which indicates high-quality read sequences and a high-quality assembled and annotated transcriptome (Table S5). After performing differential gene expression analysis using the DESeq2 package in the R environment [63], functional annotation using the EggNOG online database [65] revealed 291 genes with an increased expression level and 351 genes with a decreased expression level when *A. puulaauensis* (EXF-7678) was exposed to Lascaux 498 HV glue (Tables S6 and S7). When the fungus was exposed to the hydrocarbon resin Regalrez 1094, there were 30 upregulated genes and 41 downregulated genes (Tables S8 and S9). Using the online tool SignalP [66,67], differentially expressed genes were checked to see if their predicted proteins have signal sequences for targeting the secretory (Sec) pathway. The number of differentially expressed genes for esterases, oxidases, and oxidoreductases in each condition, together with the number of proteins of each group with a signal peptide, is listed in Table 3.

Fisher’s exact test with control of false positive results (“false discovery rate”) in the R environment exposed no gene family as significantly over- or under-represented among the differentially expressed genes when using a threshold value of 0.05.

## 4. Discussion

Canvas paintings are susceptible to biodeterioration due to their diverse chemical composition, which provides nutrients to microorganisms [70]. These works of art are constantly exposed to fungal contamination from the air [71], even in facilities with controlled environmental conditions. In recent years, research has primarily focused on the biodiversity of fungal species [24,72], the enzymatic degradation of organic compounds in canvas paintings, and the release of acidic products of microbial metabolism [25,26,27,28,29]. However, less attention has been focused on the degradation of synthetic compounds, which are considered much more stable than traditionally used materials and are increasingly used in modern art and conservation–restoration practices [73,74,75].

### 4.1. The Influence of Common Contaminants of Artistic Paintings, Facultatively Xerophilic Fungi of the Genera Aspergillus, Penicillium, and Cladosporium

The growth of fungi on synthetic materials is already a known phenomenon. The growth of fungi on alkyd resins, which have been used as pigment binders since the late 1930s, has been extensively documented. These materials are primarily biodeteriorated by species of *Aspergillus*, *Aureobasidium*, *Cladosporium*, *Gliocladium*, and *Penicillium* [17,19]. Additionally, growth of *Cenococcum*, *Eladia*, *Epicoccum*, *Glyphium*, *Phoma*, and *Talaromyces* species has been reported on acrylic resins used to restore various cultural heritage objects [6,16,17,18,19,20,21]. One study noted the growth of *Aspergillus nidulans*, *A. terreus*, *Penicillium asperum* (current name *P. kewense*), and *P. funiculosum* (current name *Talaromyces funiculosus*) on museum textiles preserved with PVA, acrylic polymers (e.g., Paraloid B72, Lascaux 360 HV, Plextol B-500, Acyloid F-10, Plexisol P-550), and BEVA 371 [76]. Moreover, *Aspergillus niger* was found to be the most common fungus on many tested acrylic and alkyd resins [17]. *Aspergillus versicolor*, *A. flavus*, *Aureobasidium pullulans*, *Chaetomium globosum*, and *Penicillium pinophilum* (current name *Talaromyces pinophilus*) also overgrew some acrylic and alkyd resins [17,77]. The deterioration of polyacrylic (by *Penicillium citrinum*) and polyurethane resins (by *Penicillium citrinum* and *Aureobasidium pullulans*) has been reported by Campana et al. (2022) [16].

The growth of fungi on different synthetic materials from other fields has also been documented [3]: *Aspergillus* species (*A. flavus*, *A. clavatus*, *A. fumigatus*) and *Pleurotus ostreatus* on vinylic polyethylene, *Aspergillus flavus*, and *Cochliobolus* sp. on PVC, *Phanerochaete chrysosporium* and *Aspergillus niger* on polypropylene, *Cephalosporium* sp., *Mucor* spp. on polystyrene, *A. fumigatus* on polyurethane, and chromofore fungi on polyamides. Moreover, synthetic paints have been biodeteriorated by *Alternaria* spp., *Aspergillus* (*A. niger* and *A. flavus*), *Aureobasidium pullulans*, *Penicillium citrinum*, *Talaromyces* species named as *Penicillium* in the original literature (*T. purpureogenus*, *T. pinophilus*, *T. variabile*), *Cladosporium* spp., *Nigrospora* spp., *Chaetomium globosum*, *Epicoccum nigrum*, and *Gliocladium virens* (current name *Trichoderma virens*).

Common contaminants of synthetic restoration materials are fungi from the genera *Aspergillus*, *Cladosporium*, and *Penicillium* [6,16,17,18,19,20,21,29]. These fungi possess numerous problematic enzymatic activities, which are potentially detrimental to painting materials [26,32]. Esterolytic, lipolytic, proteolytic, and ureolytic activities [31], including the ability to degrade hydrocarbons, are involved in degrading synthetic polymers, their additives, or their residual monomers [30,78,79]. In particular, *Aspergillus* and *Penicillium* can significantly influence materials by releasing acids into the substrate [26,27,32,37], chemically altering or degrading the substrates [30], although the impact of their enzyme secretion remains unclear.

In our study, species from the genera *Aspergillus* and *Penicillium* overgrew all tested materials. In addition to obligately xerophilic aspergilli, *A. puulaauensis* chemically altered the tested materials the most, causing changes in carbonyl bands in the spectra of hydrocarbon resin Regalrez 1094, and unaged acrylic dispersion Lascaux 498 HV. The changes in carbonyl band in FTIR spectra of hydrocarbons result from degradation processes of polymers, mainly oxidation and hydrolysis [80], where C=O bonds appear. This information is crucial as these chemical reactions can cause visual (yellowing) and mechanical changes (brittleness) in the analyzed materials over time [81]. Among others, *A. puulaauensis* displayed the most diverse set of enzymatic activities among the recently tested species [29]. It has strong esterase and urease activity, presumably contributing to the degradation of butyl methacrylate copolymers of Lascaux 498 HV.

Moreover, lipase and esterase activities are the main activities of bacteria inhabiting acrylic polymer Paraloid B72 [82]. In this study, *A. puulaauensis* displayed strong lipase activity on 4-NP butyrate (above 50 μmol min^−1^ mg^−1^), consistent with previous findings on agar plates [29]. *Aspergillus puulaauensis* is also known to assimilate mineral oil, a complex mixture of hydrocarbons [29]. The ability of many *Aspergillus* and *Pencillium* species to degrade polycyclic aromatic hydrocarbons (PAHs) can potentially contribute to the chemical degradation of hydrocarbon resin, such as Regalrez 1094 [83,84,85,86,87]. Protease activity, which could also be involved in polymer degradation [31], was not reported [29].

The growth of *Penicillium chrysogenum* induced significant changes in the carbonyl bands of aged Regalrez 1094, supported by its strong ability to assimilate hexadecane and mineral oil [29], and the ability of *Penicillium* to degrade PAHs [85,87]. *Penicillium* species are frequently found on polyurethane and acrylic resins [16]. Additionally, *P. chrysogenum* is a known producer of acids and has a diverse enzymatic profile that includes protease, urease, esterase, and lipase activities [29]. Weak lipase activity was confirmed in this study on two tested substrates: 4-NP octanoate and 4-NP palmitate.

Some fungi grew abundantly on the selected materials and did not cause any detectable changes in their chemical structure. For example, *Aspergillus pseudoglaucus* expressed esterase and lipase activity, consistent with tests on agar plates [29]. Similarly, *Penicillium corylophilum* showed abundant growth but did not cause changes in the spectra. This fungus is otherwise known for producing acids, assimilating hydrocarbons, and exhibiting urease and strong esterase activities [29]. The latter was confirmed as having high specific activity on 4-NP butyrate. It also displayed strong lipolytic activity. The growth of both species on synthetic materials is reported for the first time. Fungal enzymatic activities do not always contribute to the detectable chemical degradation of synthetic materials. In fact, fungi inoculated onto chemically demanding synthetic materials may require some time to adapt to the new complex substrate before producing key enzymes for degradation [17]. Moreover, one reason for their growth might be the presence of extracellular DNA and other nutrients from possible contaminants within materials or on their surface. All tested xerophilic species, except for *Beauveria pseudobassiana*, *Chaetomium globosum*, *Aspergillus magnivesiculatus*, and species of the genus *Wallemia*, are capable of extracellular DNase activity. Specifically, DNase activity has been reported for the obligate xerophiles *Aspergillus destruens* (EXF-7651) and *A. vitricola* (EXF-10463) [29].

The growth of *Aureobasidium pullulans*, *Chaetomium globosum*, and *Parengyodontium album* was not abundant enough to measure their influence on all selected materials by FTIR-PAS. *Aureobasidium pullulans* is a common contaminant of alkyd resins and polyurethane [16,17,88], with strong esterase, lipase, protease, and urease activities [29,88]. Strong esterase and lipase activity were also confirmed in this study. Its influence on material composition was measured only on Lascaux glues, where no chemical degradation was detected. *Chaetomium globosum* is an alkyd and acrylic resin contaminant with weak esterase activity and the ability to assimilate hydrocarbons [19,76]. Herein, it performed strong esterolytic and lipolytic activities. *Parengyodontium album* has a diverse and strong potential for degrading synthetic materials, including strong protease, esterase, lipase, and urease activities, and the ability to assimilate hydrocarbons [29]. Despite their diverse enzymatic action, neither fungus caused changes in Lascaux 498 HV, Laropal A81, or Regalrez 1094. The esterolytic and ureolytic *Wallemia canadensis* [29], which appeared on Acrylharz P550, Laropal A81, and Regalrez 1094, exhibited less abundant growth. We did not confirm its esterolytic activity, and no chemical modifications were induced in the materials.

Entomopathogenic *Beauveria pseudobassiana* and *Cladosporium cladosporioides* grew on all tested materials, but their growth was insufficient for further analysis. Fungi of the genus *Beauveria* are known contaminants of cultural heritage, but they have yet to be observed growing on synthetic polymers. *Beauveria pseudobassiana* showed esterase and urease activities on agar plates and the ability to assimilate various hydrocarbons [29]. Its esterase and lipase activity were confirmed in this study. Fungi from the genus *Cladosporium* grow well on alkyd and acrylic resins [17,18,89], and are known for their esterolytic and ureolytic activities [29], and as good degraders of PAHs [90,91]. The common contaminant of canvas paintings, *Cladosporium cladosporioides* [72], has a diverse enzymatic profile that includes protease, esterase, lipase, and urease activities, and the capacity to use hexadecane as a nutrient source [29], with strong esterase and lipase activities also confirmed in this study.

### 4.2. Some Obligately Xerophilic Fungi with a Narrow Spectrum of Enzymatic Activities Can Chemically Modify Synthetic Materials

Obligately xerophilic fungi of the genus *Aspergillus* are common contaminants of canvas paintings [39,92]. Several recent studies have focused on this group of fungi [93] and their degradation potential [29,39]. However, these studies were not related to the degradation of synthetic compounds used in the conservation–restoration of artworks [29]. The obligate xerophiles *A. destruens* and *A. vitricola* are the most common xerophilic species on canvas paintings [39]. Tolerance to low water activity is also known for species of the genus *Penicillium*, such as *P. chrysogenum*, which can germinate at very low water activities (a_w_ 0.78) [47], and *Cladosporium*, where some species (*C. sphaerospermum* and *C. halotolerans*) can grow at a_w_ as low as 0.82 [94]. The genus *Wallemia*, physiologically unique among the Basidiomycota, is commonly found at low relative humidity and grows at a_w_ > 0.75 [47].

The most noticeable changes in the chemical structure of the selected materials among obligately xerophilic fungi were caused by *Aspergillus destruens*, which affected the carbonyl bands of Regalrez 1094 and Lascaux 498 HV, and the C-H bonds of Laropal A81. The impact of *A. destruens* on materials is consistent with its abundant growth on all materials, its strong esterase activity observed on agar plates [29], and its ability to degrade hydrocarbons under saline conditions [84]. However, we did not confirm its esterase activity in this study, detecting only weak lipase activity. This discrepancy could be attributed to the different substrates used in agar plate tests: Tween-80 for esterase activity and tributyrin for lipase activity.

The obligate xerophilic fungus *A. vitricola* caused changes in the carbonyl bands of Regalrez 1094, aged Lascaux 498 HV, and in carbonyl bands and C-H bonds of aged Laropal A81. This fungus is not known for any enzymatic activities potentially involved in the degradation of synthetic polymers [29]. In this study, it exhibited very weak lipase activity.

*Aspergillus magnivesiculatus*, another common obligate xerophile, modified the carbonyl bands of Regalrez 1094 and non-aged Lascaux 498 HV. It is known for its amylase, cellulase, urease, and DNase activities and its ability to degrade hexadecane, distinguishing it from other xerophiles as a significant threat to canvas paintings [29]. However, we measured very weak esterase and lipase activities (below 10 μmol min^−1^ mg^−1^), which is not by previously reported tests on agar plates [29].

*Wallemia* sp. (aff. *muriae*), also considered a significant threat to canvas paintings, did not grow sufficiently to provide detailed information on changes in the chemical structure of the materials. It is known only for its urease activity [29], and no esterase or lipase activity was measured in this study.

### 4.3. The Restoration Materials Most Susceptible to the Effects of Fungal Growth Are Laropal A81, Regalrez 1094, and Acrylic Dispersion Lascaux 498 HV

The most pronounced alterations in the chemical structure of the tested substrates were detected in the spectra of urea–aldehyde resin Laropal A81 (changes in carbonyl band and C-H bonds) and hydrocarbon resin Regalrez 1094 (carbonyl bands), followed by acrylic dispersion Lascaux 498 HV (carbonyl bands). Fewer changes were observed in Acrylharz P550, BEVA 371, and Lascaux 303 HV, primarily indicated by the change in water and amide bands.

Acrylic polymers are relatively stable and resistant materials, commonly used as binders for paints, adhesives, and surface consolidants. Frequently used acrylic materials include polymethacrylates, often employed as consolidants and for the protection of cultural heritage [95]. Some acrylic polymers, such as the well-studied synthetic product Paraloid B72, are considered less suitable due to their tendency to undergo photo-oxidation reactions, typically induced by sunlight, temperature, and atmospheric pollution, leading to altered mechanical characteristics [96]. In this study, we tested three acrylic materials based on butyl methacrylate: two Lascaux dispersions and acrylic resin Acrylharz P550. As expected, Acrylharz P550 resin was the most resistant to chemical changes [4,96]. However, none of the tested acrylic materials were entirely resistant to fungal growth. It is also important to note that both unaged and artificially aged materials were similarly chemically affected after the fungal inoculation, with the exception of both Lascaux glues, which were more affected when artificially aged.

Among all the materials used in the study, Lascaux 303 HV was one of the least susceptible to chemical degradation, in contrast to Lascaux 498 HV, which was one of the most susceptible to chemical changes. The behavior of products within the same class of polymers can vary, likely due to the presence of other compounds, especially in dispersions [5]. Even copolymers and monomers of one polymer can behave differently, with monomers being less susceptible to biodeterioration [97]. By previous research, it is likely that fungi were able to grow on Lascaux 498 HV due to the presence of additives [17], which can significantly diminish the ability of water-based dispersions to withstand water, heat, and mechanical stress [98].

Laropal A81 is known as a pigment binder [99] and is one of the most stable resins currently used as a varnish for paintings [100,101]. Regalrez 1094, a hydrocarbon resin resembling natural resins, is widely used in art restoration for its chemical stability and low-molecular weight [6,44], even after aging [102,103]. Both Laropal A81 and Regalrez 1094 have good consolidation properties for easel paintings [104] and other artistic works [105], despite being susceptible to brown-rot or white-rot fungi. In our study, we observed their similar susceptibility to fungal growth before and after UV aging. However, the FTIR PAS spectra revealed changes in the carbonyl bands of both and C-H bonds of Laropal A81, suggesting potential susceptibility to degradation by xerophilic fungi.

Additionally, SEM analyses showed deformation of the Regalrez 1094 layer after excessive growth of *Aspergillus puulaauensis*, although the fungus did not penetrate the material. Regalrez 1094 has unsaturated bonds in its structure, which can lead to changed chemical characteristics due to radical reactions [106]. On the other hand, no changes in the microstructure of Laropal A81 were observed.

BEVA 371, commonly used as a general consolidating adhesive, blends several components, including two poly(ethylene-*co*-vinyl acetates), two low-molecular-weight resins, and a wax [44,107]. Despite the growth of most of the tested fungi on BEVA 371, it remained one of the least chemically altered materials, with changes noted only in amide bonds and water vibrations. This is consistent with previous studies [76]. The presence of amide bands likely originates from the fungi on the surface of the analyzed materials [108], as hyphae were not removed before the analysis. The relatively high water content is associated with the appearance of amide bonds, likely due to water in the fungal hyphae, rather than water bound to polymers after breaking intermolecular bonds between polymer macromolecules.

Observation of the microstructure revealed fungal growth on the surface of the materials, but hyphae did not penetrate any of the tested materials, which is consistent with previous studies [76]. Complete degradation of the positive control skin glue, a protein material critical to canvas paintings [39], was observed. For the first time, the thinning of the Lascaux adhesive layer under the hyphae and the deformation of the Regalrez 1094 layer after excessive fungal growth were observed. Additionally, small bubbles were visible in Acrylharz P550, likely due to the evaporation of solvents or volatile products [6].

Fungal growth can also occur due to chemically diverse additives, such as plasticizers, stabilizers, antistatic agents, and lubricants [30]. Acrylic materials may contain other acrylic monomers, such as styrene, vinyl chloride, ethylene, and vinyl acetate, increasing their susceptibility to fungal attack [17]. Furthermore, different resins are often found on artworks in overlapping layers for greater stability, which can serve as nutrient sources [6]. Additionally, fungal growth on the remains of dead microorganisms or insects on the surface of the polymers can occur, with catabolic products from one microorganism serving as a nutrient source for another [16].

While it is often believed that controlled temperature and humidity should prevent fungal growth on canvas paintings and that contemporary synthetic materials are safe from microbial degradation, the results of this work and other recent studies indicate this is not the case [29,39,109]. Xerophilic fungi can grow at very low humidity and can accelerate the deterioration of some often-used synthetic materials. The findings on the biology of these fungi and the large differences in the resilience of various synthetic materials provide essential clues for successful long-term preservation and restoration of valuable artworks.

This study highlights the complex interactions between fungi and synthetic materials, emphasizing the need for further research to develop practical and effective conservation guidelines and strategies for artworks using synthetic restoration materials.

### 4.4. Analysis of the Transcriptome of Aspergillus puulaauensis Indicates Ability to Degrade Restoration Materials by Oxidases and Esterases

Transcriptome analysis of the fungus *Aspergillus puulaauensis* (EXF-7678) grown on acrylic dispersion Lascaux 498 HV and hydrocarbon resin Regalrez 1094 revealed fewer differentially expressed genes on Regalrez 1094 than on Lascaux 498 HV, suggesting differences in the composition of the two materials. The acrylic dispersion is partially water-soluble, making it more accessible to microorganisms, unlike the more rigid Regalrez 1094 resin. In both cases, we observed increased expression of genes encoding oxidases and esterases, which may indicate their (non)specific roles in the biochemical degradation of these materials.

Transcriptome analysis of the fungus grown on synthetic materials revealed characteristically altered (both increased and decreased) gene expression levels for some peroxidases, oxidases, and oxidoreductases. In the presence of acrylic glue Lascaux 498 HV, the fungus exhibited increased expression of genes encoding oxidases, which include xylan, cellulose, and chitin monooxygenases. Some of them can potentially degrade plastic polymers [110]. Altered expression of peroxidase genes could be associated with the response to oxidative stress [86,111]. Within this enzymatic group, manganese and lignin peroxidases are also present, both of which are known to degrade certain synthetic polymers [112,113].

Transcriptome analysis revealed both increased and decreased expression levels of genes encoding acetyl xylan esterases in the presence of hydrocarbon resin Regalrez 1094, although these enzymes do not participate in plastic degradation. In contrast, in the presence of Lascaux glue 498 HV, the fungus exhibited altered expression of several genes encoding esterases, including a reduction in the expression of the cutinase gene. Cutinases are known to hydrolyze various synthetic polyesters, such as polycaprolactone [114], polyethylene terephthalate [115], and polytrimethylene terephthalate [116]. Additionally, using the online tool SignalP, we identified differentially expressed genes encoding esterases whose predicted proteins contain signal sequences for targeting the secretory (Sec) pathway, enabling their activity outside the cell. These include enzymes involved in the degradation of synthetic polymers, such as feruloyl esterase [117], carboxyl ester hydrolase [118], and cutinases [119].

Fungi can modulate the expression of genes encoding esterases and oxidases when grown on modern synthetic acrylic materials. This dual regulation likely results from the complex interaction between metabolic adaptation and environmental signaling. Furthermore, it is important to note that comparisons between transcriptome analyses and measured enzyme activity can only be partially informative. Enzyme activity may also be influenced by the use of specific substrate used, experimental conditions, the efficiency of enzyme secretion from cells, and their activity within the extracellular environment. Interactions between fungi and synthetic materials are complex. Nevertheless, as the first study to analyze the transcriptome in the context of conservation–restoration, these findings are intriguing and lay a valuable foundation for further research. Future studies will need to explore the underlying mechanisms of fungal adaptation to synthetic materials and provide new insights into the preservation of these materials.

## 5. Conclusions

This study shows that synthetic materials used in canvas painting restoration, including acrylic dispersions (Lascaux 498 HV, Lascaux 303 HV), acrylic resin (Acrylharz P550), urea–aldehyde resin (Laropal A81), hydrocarbon resin (Regalrez 1094), and epoxy/vinyl acetate blend (BEVA 371), are susceptible to biodeterioration by xerophilic fungi. *Aspergillus* and *Penicillium* species, particularly *A. vitricola*, *A. destruens*, *A. puulaauensis*, and *P. chrysogenum*, extensively colonize these materials. They induce chemical changes, such as alterations in carbonyl and C-H bonds, leading to long-term visual and mechanical deterioration, including yellowing and brittleness. These findings indicate that while materials such as BEVA 371, Acrylharz P550, and Lascaux 303 HV exhibit initial resistance to fungal colonization, their susceptibility increases significantly after artificial aging. This suggests that their long-term stability is compromised, raising concerns about their suitability for the durable conservation and restoration of cultural heritage objects.

Notably, *Aspergillus puulaauensis* causes significant chemical alterations to Lascaux glues and Regalrez 1094. The transcriptomic analysis revealed changes in the regulation of genes encoding esterases and oxidases, enzymes also involved in synthetic polymer degradation. However, given the vast number of potential enzymes contributing to material deterioration, the results provide a foundation for future studies.

These findings demonstrate that synthetic materials commonly used in the restoration and conservation of artworks are vulnerable to biodeterioration by xerotolerant/xerophilic fungi—an understudied group often excluded from conventional biodeterioration assessments and material testing protocols. The increased susceptibility observed in artificially aged samples of Laropal A81, Regalrez 1094, and Lascaux glues (303, 498 HV) highlights the progressive degradation of these polymers over time. Addressing this issue requires a deeper understanding of the chemical changes occurring in synthetic materials, as well as the metabolic pathways employed by xerophilic fungi in their colonization and breakdown. Such knowledge is essential to support informed decision-making in conservation practice and to guide the selection of materials with greater long-term stability.

## Figures and Tables

**Table 2:** Growth of xerophilic fungi on synthetic materials after 2 months of incubation at 90% RH. Obligate xerophyles (shown in bold) were incubated for 2 months at 70% RH and 5 months at 90% RH.

		Skin Glue		Lascaux 303		Lascaux 498		Acrylharz P550		Laropal A81		Beva 371		Regalrez 1094	
EXF- ^a^	Species	N	A	N	A	N	A	N	A	N	A	N	A	N	A
10360	***Aspergillus destruens***	+++	+++	++	++	+	+++	+	+	+	+	+	+	+	+
10353	***Aspergillus*** ***magnivesiculatus***	+++	+++	−	++	+	+	+	+	+	+	+	+	+	+
14316	*Aspergillus* *pseudoglaucus*	**++**	**+++**	−	+	**−**	**+**	+	+	**+**	**++**	−	+	**+**	**++**
7678	*Aspergillus* *puulaauensis*	**+++**	**+++**	−	+	**−**	**+**	++	++	**+**	**++**	+	+	**++**	**++**
15210	***Aspergillus*** ***vitricola***	+++	+++	−	+	−	++	+	+	+	+	+	+	+	+
14317	*Aureobasidium* *pullulans*	**++**	**++**	−	−	**−**	**−**	−	−	**−**	**−**	−	−	**−**	**−**
10556	*Beauveria* *pseudobassiana*	**++**	**+++**	−	+	**−**	**+**	−	+	**+**	**+**	+	+	**+**	**+**
7690	*Chaetomium* *globosum*	**+++**	**+**	−	−	**−**	**+**	−	−	**−**	**+**	−	−	**−**	**−**
14315	*Cladosporium cladosporioides*	**++**	**+++**	−	+	**−**	**−**	−	+	**+**	**+**	+	+	**+**	**+**
10663	*Parengyodontium album*	**++**	**+++**	−	−	**−**	**+**	−	−	**−**	**+**	−	−	**−**	**+**
10495	*Penicillium* *chrysogenum*	**+++**	**+++**	−	+	**+**	**−**	+	+	**+**	**+**	+	+	**+**	**+**
15064	*Penicillium* *corylophilum*	**++**	**++**	−	++	**−**	**++**	+	−	**+**	**+**	+	++	**+**	**+**
10120	***Wallemia* sp. (aff.** ***muriae*)**	+++	+++	−	−	−	−	−	−	−	−	−	−	−	−
10342	*Wallemia* *canadensis*	**++**	**++**	−	−	**−**	**−**	+	+	**+**	**+**	−	−	**+**	**+**

Legend: Growth was evaluated visually and scored based on the extent of mycelium development using the following criteria: (−) no visible growth; (+) weak growth characterized by the presence of hyphae covering less than half of the inoculated area; (++) moderate growth, where mycelium covered more than half of the inoculated area (5 mm diameter); and (+++) strong growth, where mycelium fully covered the inoculated spot and extended onto the surrounding glass surface. N—non-aged; A—aged. ^a^ EXF—designation of fungal strains from the Ex Culture Collection of the Infrastructural Centre Mycosmo, MRIC UL, Slovenia (Department of Biology, Biotechnical Faculty, University of Ljubljana).

**Table 3:** Number of differentially expressed genes for esterases, oxidases, and oxidoreductases of the fungus *Aspergillus puulaauensis* in the presence of Lascaux 498 HV or Regalrez 1094.

	UP (Lascaux 498 HV)	DOWN (Lascaux 498 HV)	UP (Regalrez 1094)	DOWN (Regalrez 1094)
Esterase	9	16	2	3
Esterase SP	4	11	1	3
Oxidase	15	22	1	3
Oxidase SP	2	6	0	2
Oxidoreductase	12	16	1	4
Oxidoreductase SP	1	3	0	0

Legend: SP—signal peptide, DOWN—downregulated, UP—upregulated.

## Data Availability

Data are contained within the article and the Supplementary Materials.

## Supplementary Materials

The following supporting information can be downloaded at: https://www.mdpi.com/article/10.3390/jof11080568/s1, Table S1: cultures used in this study, their source, and identification; Table S2: overview of FT-IR PAS spectra changes in skin glue, Lascaux 303 HV, Lascaux 498 HV, Acrylharz P550, Laropal A81, Beva 371, and Regalrez 1094, infected by xerotolerant fungi; Table S3: overview of FT-IR PAS spectra changes in skin glue, Lascaux 303 HV, Lascaux 498 HV, Acrylharz P550, Laropal A81, Beva 371, and Regalrez 1094, infected by obligately xerophilic fungi; Table S4: esterolytic activity of tested fungal species measured on 4-nitrophenyl acetate, 4-nitrophenyl butyrate, 4-nitrophenyl octanoate, and 4-nitrophenyl palmitate; Table S5: results of the quality analysis of the de novo assembled and annotated transcriptome of the fungus *Aspergillus puulaauensis* (EXF-7678) with the BUSCO program for the dataset “fungi_odb10”. Proportions of a total of 758 searched genes that are universally (and in single copy) present in fungal genomes are shown. Table S6: results of the differentially expressed genes (increased expression) analysis of the fungus *Aspergillus puulaauensis* (EXF-7678) grown on synthetic material Lascaux 498 obtained using the EggNOG online database; Table S7: results of differentially expressed genes (reduced expression) analysis of the fungus *Aspergillus puulaauensis* (EXF-7678) grown on synthetic material Lascaux 498 obtained using the EggNOG online database; Table S8: results of the differentially expressed genes (increased expression) analysis of the fungus *Aspergillus puulaauensis* (EXF-7678) grown on synthetic material Regalrez 1094, obtained using the EggNOG online database; Table S9: results of the differentially expressed genes (reduced expression) analysis of the fungus *Aspergillus puulaauensis* (EXF-7678) grown on synthetic material Regalrez 1094, obtained using the EggNOG online database; Figure S1: esterase activities of selected fungi, expressed in μmol of 4-nitrophenyl (4-NP) substrate/mg of fungal supernatant.

## Abbreviations

The following abbreviations are used in this manuscript:

EXF	designation of fungal strains from the Ex Culture Collection of the Infrastructural Centre Mycosmo, MRIC UL, Slovenia (Department of Biology, Biotechnical Faculty, University of Ljubljana)
FTIR-PAS	Fourier-transform infrared photoacoustic spectroscopy
SEM	Scanning electron microscopy
