# Supplementary material for: Degradation of Synthetic Restoration Materials by Xerotolerant/Xerophilic Fungi Contaminating Canvas Paintings
Source: J Fungi (Basel). 2025 Jul 30;11(8):568. doi: 10.3390/jof11080568 (PMC12387268; doi:10.3390/jof11080568)

Supplementary Material

Degradation of Synthetic Restoration Materials by Xerotolerant/Xerophilic Fungi Contaminating Canvas Paintings

Amela Kujović[1], Katja Kavkler[2], Michel Alexander Wilson-Hernandez[3], Miloš Vittori[1], Luen Zidar[1], Cene Gostinčar[1], Kristina Sepčić[1], Yordanis Pérez-Llano[3], Ramón Alberto Batista-García[3], Nina Gunde-Cimerman[1], Polona Zalar[1,*]
*** Correspondence:** Polona Zalar: polona.zalar@bf.uni-lj.si

1 Supplementary Tables

Table S1: Cultures used in this study, their source and identification. The selected fungal strains, tested for their ability to degrade synthetic materials, were selected based on strong enzymatic and other activities [29], and their frequency of isolation from canvas paintings [39, 109].

EXF strain nr.	Fungal name	Source	Support	Paint	Isolation side	No. of paintings from which was isolated	Enzyme activities	Identification DNA barcode: GenBank No.	Reference
10360	*Aspergillus destruens*	painting RCS 25	flax canvas	oil	V	11	ES	*BenA*: MW369690	Zalar et al. 2023 [39]
10353	*Aspergillus magnivesiculatus*	painting RCS 24	flax canvas	oil	V	9	URE	*BenA*: MW357145	Zalar et al. 2023 [39]

EXF strain nr.	Fungal name	Source	Support	Paint	Isolation side	No. of paintings from which was isolated	Enzyme activities	Identification DNA barcode: GenBank No.	Reference
14316	*Aspergillus pseudoglaucus*	mock up sample contamination	wood impregnated with animal glue, oil paint, and Regalrez 1126	oil	V	5	ES, LIP, LEC	*BenA*: PP034128	Kujović et al., 2024 [29]
7678	*Aspergillus puulaauensis*	painting RCS 19	flax canvas	oil	R	2	ES, LEC, URE, MO	*BenA*: MW357120	Zalar et al. 2023 [39]
15210	*Aspergillus vitricola*	painting RCS 22	hemp canvas	oil or greasy tempera	R	19	-	ITS: PP033020	Zalar et al. 2023 [39]
14317	*Aureobasidium pullulans*	mock up sample contamination	wood, animal glue, oil paint, Beva 371	oil	V	1	ES, LIP, LEC, URE, GEL	ITS: PP033021	Kujović et al., 2024 [29]
10556	*Beauveria pseudobassiana*	painting GBJ 2	unknown canvas	oil	V	2	URE, HCA, LAC	ITS: MW288719	Zalar et al. 2023 [39]
7690	*Chaetomium globosum*	painting RCS 18	hemp canvas	oil	R	1	URE, HEX	ITS: MW288933	Zalar et al. 2023 [39]
14315	*Cladosporium cladosporioides*	mock up sample contamination	glass impregnated with Regalrez 1126	-	-	2	ES, LEC, URE, CAS, LAC	*act*: PP034131	Kujović et al., 2024 [29]
10663	*Parengyodontium album*	Celje ceiling	linen canvas	tempera	V	1	ES, LIP, LEC, URE, HCA, CAS	ITS: MZ687373	Kavkler et al. 2022 [109]
10495	*Penicillium chrysogenum*	painting GBJ 2	unknown canvas	oil	V	10	LEC, URE, HCA, GEL	*BenA*: MW357233	Zalar et al. 2023 [39]

EXF strain nr.	Fungal name	Source	Support	Paint	Isolation side	No. of paintings from which was isolated	Enzyme activities	Identification DNA barcode: GenBank No.	Reference
15064	*Penicillium corylophilum*	painting ART-1	flax canvas	oil	V	3	ES, LEC, URE, HCA	*BenA*: MW357214	Zalar et al. 2023 [39]
10120	*Wallemia* sp. (aff. *muriae*)	painting RCS 20	flax canvas	oil and greasy tempera	V	6	URE	ITS: MW288065	Zalar et al. 2023 [39]
10342	*Wallemia canadensis*	painting RCS 21	flax canvas	oil and greasy tempera	R	2	ES, URE	ITS: MW288071	Zalar et al. 2023 [39]

Legend: GM: Regional Museum Goriški muzej, Solkan (Slovenia); GB: Božidar Jakac Art Museum, Kostanjevica na Krki (Slovenia); RCS: Restoration Centre, Institute for the Protection of Cultural Heritage of Slovenia, Ljubljana (Slovenia); ART-1: painting in church, Koper (Slovenia); ART-4: painting in church, Leskovec at Višnja gora (Slovenia). V: isolated from the verso (front side) of the painting; R: isolated from the recto (back side) of the painting. ITS, internal transcribed spacer region including intervening 5.8S rRNA gene; *act*, partial actin gene; *tef1*, partial translation elongation factor 1-alpha gene; *BenA*, partial beta-tubulin gene; ES: esterase; LEC: lecithinase; LIP: lipase; URE: urease; HCA: hydrocarbon assimilation (HEX: hexadecane, MO: mineral oil); LAC: laccase; CAS: caseinase; GEL: gelatinase. EXF - designation of fungal strains in the Ex Culture Collection of the Infrastructural Centre Mycosmo, MRIC UL, Slovenia (Department of Biology, Biotechnical Faculty, University of Ljubljana). For more information on paintings we refer to Kavkler et al. (2022), Zalar et al. (2023), and Kujović et al. (2024).

Table S2: Overview of FTIR PAS spectra changes of skin glue, Lascaux 303 HV, Lascaux 498 HV, Acrylharz P550, Laropal A81, Beva 371, and Regalrez 1094, infected by xerotolerant fungi

	EXF	Fungus/Material	Skin glue	Lascaux 303 HV	Lascaux 498 HV	Acrylharz P550	Laropal A81	BEVA 371	Regalrez 1094
C			0	0	0	0	water stretching band/ increase, carbonyl band/ shift to lower wavenumbers, shoulders 1680 and 1630 cm-1, decrease 1443 cm-1	C-H vibrations/ changes, increase of shoulder 1630 cm-1, 1650 cm-1/ decrease	carbonyl band/ appearence
N	**14316**	***Aspergillus pseudoglaucus***	0	0	0	0	0	0	0
	**7678**	***Aspergillus puulaauensis***	carbonyl band/ shoulder, amide bands/ shift	0	carbonyl band/ slight decrease, disappearing 1541 and 1575 cm-1, possibly fungi	water increase, amide bands/ fungi	water stretching band/ increase	increase of shoulder 1630 cm-1	appearence 1630 cm-1 (water or fungi), carbonyl band/ shoulder
	**14317**	***Aureobasidium pullulans***	carbonyl band/ shoulder	0	0	/	/	/	/
	**10556**	***Beauveria pseudobassiana***	/	/	/	/	/	/	/
	**7690**	***Chaetomium globosum***	0	/	0	/	0	/	0
	**14315**	***Cladosporium cladosporioides***	/	/	/	/	/	/	/
	**10663**	***Parengyodontium album***	0	/	0	/	0	/	0
	**10495**	***Penicillium chrysogenum***	/	amide bands/ some points	/	0	0	0	0
	**15064**	***Penicillium corylophilum***	0	0	0	0	0	0	0
	**10342**	***Wallemia canadensis***	0	/	0	0	0	0	0
A	**14316**	***Aspergillus pseudoglaucus***	0	0	0	0	0	0	0
	**7678**	***Aspergillus puulaauensis***	carbonyl band/ shoulder, amide bands/ shift	water stretching band/ increase; amide vibrations	water stretching band/ increase, shoulder 1120	water increase, amide bands/ fungi	0	water stretching band/ increase, shoulder 1630 cm-1	water stretching band/ increase, carbonyl band/ shift to higher wavenumbers,

					cm-1, possibly fungi				appearance 1645 cm-1 (water or fungi)
	**14317**	***Aureobasidium pullulans***	carbonyl band/ shoulder	0	0	/	/	/	/
	**10556**	***Beauveria pseudobassiana***	/	/	/	/	/	/	/
	**7690**	***Chaetomium globosum***	0	/	0	/	0	/	0
	**14315**	***Cladosporium cladosporioides***	/	/	/	/	/	/	/
	**10663**	***Parengyodontium album***	0	/	0	/	0	/	0
	**10495**	***Penicillium chrysogenum***	/	0	/	0	0	0	carbonyl band/ decrease
	**15064**	***Penicillium corylophilum***	amide bands/ shift	0	0	0	0	0	0
	**10342**	***Wallemia canadensis***	0	/	0	0	0	0	0

Legend: 0 – no changes; / – not measured; C – control; N – freshly applied material (non-aged); A – artificially aged material; EXF - designation of fungal strains in the Ex Culture Collection of the Infrastructural Centre Mycosmo, MRIC UL, Slovenia (Department of Biology, Biotechnical Faculty, University of Ljubljana). For more information on paintings we refer to Zalar et al. (2023). and Kujović et al. (2024).

References:

[29]* Kujović, A., Gostinčar, C., Kavkler, K., Govedić, N., Gunde-Cimerman, N., & Zalar, P. (2024). Degradation Potential of Xerophilic and Xerotolerant Fungi Contaminating Historic Canvas Paintings. Journal of fungi (Basel, Switzerland), 10(1), 76. doi:10.3390/jof10010076

[39] Zalar, P.; Graf Hriberšek, D.; Gostinčar, C.; Breskvar, M.; Džeroski, S.; Matul, M.; Novak Babič, M.; Čremožnik Zupančič, J.; Kujović, A.; Gunde-Cimerman, N.; et al. Xerophilic Fungi Contaminating Historically Valuable Easel Paintings from Slovenia. Front. Microbiol. 2023, 14, doi:10.3389/fmicb.2023.1258670.

[109] Kavkler, K.; Humar, M.; Kržišnik, D.; Turk, M.; Tavzes, Č.; Gostinčar, C.; Džeroski, S.; Popov, S.; Penko, A.; Gunde - Cimerman, N.; et al. A Multidisciplinary Study of Biodeteriorated Celje Ceiling, a Tempera Painting on Canvas. Int. Biodeterior. Biodegradation 2022, 170, 105389, doi:10.1016/j.ibiod.2022.105389.

* the numbers refer to the order of references in the main text of the article

Table S3. Overview of FT-IR PAS spectra changes of skin glue, Lascaux 303 HV, Lascaux 498 HV, Acrylharz P550, Laropal A81, Beva 371, and Regalrez 1094, infected by obligately xerophilic fungi.

	EXF	Fungus/Material	Skin glue	Lascaux 303 HV	Lascaux 498 HV	Acrylharz P550	Laropal A81	BEVA 371	Regalrez 1094
**CA**			0	carbonyl band/ slight increase	0	0	carbonly band/ shift to lower wavenumbers	new band (1680 cm-1)	carbonyl band/ appearence
**N**	**10360**	***Aspergillus destruens***	carbonyl band/ shoulder, amide bands/ shift to higher wavenumbers	0	0	water stretching band/ increase, amide bands/ fungi	water stretching band/ slight increase, carbonyl band/ shift to higher wavenumbers, carbonyl band/ slight increase, C-H vibrations/ changes, amide bands/ fungi	water stretching band/ increase, amide bands/ fungi	water stretching band/ increase, carbonyl band/ appearence, amide bands/ fungi
	**10353**	***Aspergillus magnivesiculatus***	amide bands/ decrease/ only with 70% RH	0	carbonyl band/ decrease	water stretching band/ increase; amide bands	water stretching band/ increase/ only at 70% RH; amide bands/ at 70% RH	water stretching band/ increase/ only at 70% RH; amide bands/ at 70% RH	carbonyl band/ appearence; water stretching band/ appearence; amide bands
	**15210**	***Aspergillus vitricola***	carbonyl band/ shoulder	0	0	water stretching band/ increase, amide bands/ fungi	water stretching band/ increase, amide bands/ fungi?	water stretching band/ increase, amide bands/ fungi	water stretching band/ increase, carbonyl band/ appearence, amide bands/ fungi
	**10120**	***Wallemia* aff. *muriae***	0	(no analysis)	(no analysis)	(no analysis)	(no analysis)	(no analysis)	(no analysis)
**A**	**10360**	***Aspergillus destruens***	carbonyl band/ shoulder	water stretching band/ increase, amide bands/ fungi	water stretching band/increase	water stretching band/ increase, amide bands/ fungi	water stretching band/ increase, C-H vibrations/ changes, carbonyl band/ shift to higher wavenumbers, amide bands/ fungi	water stretching band/ increase, amide bands/ fungi?	water stretching band/ increase, carbonyl band/ appearence, amide bands/ fungi

	**10353**	***Aspergillus magnivesiculatus***	amide bands/ decrease7 more intensive at 50% RH	water stretching bands/ increase, amide bands/ fungi	water stretching band/ increase; amide bands; bands 1240 and 1065 cm-1/ decrease	water stretching band/ increase; amide bands	water stretching band/ increase/ only at 70% RH; amide bands/ at 70% RH	water stretching band/ increase/ only at 70% RH; amide bands/ at 70% RH	carbonyl band/ increase; water stretching band/ appearence; amide bands
	**15210**	***Aspergillus vitricola***	carbonyl band/ shoulder	water stretching band/ increase, amide bands/ fungi	water stretching band/ increase, carbonyl band/ slight decrease, amide bands/ fungi	0	water stretching band/ increase, C-H vibrations/ changes, amide bands/ fungi	0	water stretching band/ increase, carbonyl band/ appearence, amide bands/ fungi
	**10120**	***Wallemia aff. muriae***	0	(no analysis)	(no analysis)	(no analysis)	(no analysis)	(no analysis)	(no analysis)

Legend: 0 – no changes; / – not measured; C – control; N – freshly applied material (non-aged); A – artificially aged material; EXF - designation of fungal strains in the Ex Culture Collection of the Infrastructural Centre Mycosmo, MRIC UL, Slovenia (Department of Biology, Biotechnical Faculty, University of Ljubljana). For more information on paintings we refer to Zalar et al. (2023). and Kujović et al. (2024).

Table S4: Esterolytic activity of tested fungal species measured on 4-nitrophenyl acetate, 4-nitrophenyl butyrate, 4-nitrophenyl octanoate and 4-nitrophenyl palmitate.

**EXF**	**Genus**	**Species**	**SA 4-NP acetate [µmol min^{-1} mg^{-1}]**	**SA 4-NP butyrate [µmol min^{-1} mg^{-1}]**	**SA 4-NP octanoate [µmol min^{-1} mg^{-1}]**	**SA 4-NP palmitate [µmol min^{-1} mg^{-1}]**
14317	*Aureobasidium*	*pullulans*	71,879	165,176	135,559	44,771
14317	*Aureobasidium*	*pullulans*	44,174	149,281	94,058	32,018
14317	*Aureobasidium*	*pullulans*	68,487	179,595	129,316	47,302
10556	*Beauveria*	*pseudobassiana*	0,000	45,021	50,093	19,204
10556	*Beauveria*	*pseudobassiana*	7,560	51,881	59,647	54,037
10556	*Beauveria*	*pseudobassiana*	8,241	45,175	58,742	24,572
7690	*Chaetomium*	*globosum*	61,995	109,405	103,128	45,907
7690	*Chaetomium*	*globosum*	23,085	109,721	73,839	30,429
7690	*Chaetomium*	*globosum*	29,560	144,121	84,256	59,838
10663	*Parengyodontium*	*album*	15,779	151,572	65,638	19,753
10663	*Parengyodontium*	*album*	16,367	212,728	90,200	23,625
10663	*Parengyodontium*	*album*	18,406	179,875	56,389	23,760
14315	*Cladosporium*	*cladosporioides*	22,120	146,918	74,643	111,177
14315	*Cladosporium*	*cladosporioides*	24,454	170,835	91,691	45,053
14315	*Cladosporium*	*cladosporioides*	27,159	202,509	89,013	24,837
**15210**	***Aspergillus***	***vitricola***	18,131	5,916	7,663	12,105
**15210**	***Aspergillus***	***vitricola***	3,341	1,693	15,929	12,970
**15210**	***Aspergillus***	***vitricola***	24,876	1,355	19,683	12,857
**10360**	***Aspergillus***	***destruens***	3,120	0,000	12,275	7,529
**10360**	***Aspergillus***	***destruens***	38,364	2,409	28,055	4,183
**10360**	***Aspergillus***	***destruens***	1,951	0,342	19,959	10,558
**10353**	***Aspergillus***	***magnivesiculatus***	20,549	16,688	1,647	11,000
**10353**	***Aspergillus***	***magnivesiculatus***	2,277	0,897	1,169	8,908
**10353**	***Aspergillus***	***magnivesiculatus***	17,918	0,000	3,095	18,644

7678	*Aspergillus*	*puulaauensis*	12,143	21,044	61,924	16,468
7678	*Aspergillus*	*puulaauensis*	15,306	20,901	68,017	15,934
7678	*Aspergillus*	*puulaauensis*	11,108	19,892	46,487	32,346
14316	*Aspergillus*	*pseudoglaucus*	9,482	8,495	11,856	7,184
14316	*Aspergillus*	*pseudoglaucus*	4,116	9,843	12,144	6,235
14316	*Aspergillus*	*pseudoglaucus*	6,739	8,301	13,092	8,891
10495	*Penicillium*	*chrysogenum*	7,310	30,900	43,992	19,182
10495	*Penicillium*	*chrysogenum*	10,304	5,546	26,790	15,280
10495	*Penicillium*	*chrysogenum*	7,636	22,445	37,160	26,295
15064	*Penicillium*	*corylophilum*	17,647	96,600	57,026	14,981
15064	*Penicillium*	*corylophilum*	10,909	134,270	22,025	26,293
15064	*Penicillium*	*corylophilum*	16,581	167,560	90,979	19,444
**10120**	***Wallemia***	**sp. (aff. *muriae*)**	4,705	0,000	7,875	0,000
**10120**	***Wallemia***	**sp. (aff. *muriae*)**	5,658	8,991	4,410	13,838
**10120**	***Wallemia***	**sp. (aff. *muriae*)**	0,000	5,940	4,192	0,000
10342	*Wallemia*	*canadensis*	7,314	0,000	9,469	15,132
10342	*Wallemia*	*canadensis*	6,161	0,000	17,955	10,092
10342	*Wallemia*	*canadensis*	5,451	0,000	6,306	8,842

Legend: SA: specific activity; 4NP: 4-nitrophenol

Table S5: Results of the quality analysis of the *de novo* assembled and annotated transcriptome of the fungus *Aspergillus puulaauensis* (EXF-7678) with the BUSCO program for the dataset "fungi_odb10". Proportions of a total of 758 searched genes that are universally (and in single copy) present in fungal genomes are shown.

Genome	Complete [%]	Single-copy [%]	Duplicated [%]	Fragmented [%]	Missing [%]	Total no. of BUSCO groups
EXF-7678	99,3	92,2	7,1	0,3	0,4	758

Table S6: Results of the differentially expressed genes analysis of the fungus *Aspergillus puulaauensis* (EXF-7678) grown on synthetic material Lascaux 498 obtained using the EggNOG online database. Genes for esterases and oxidases with increased expression levels are shown. The orange color represents the group of esterases, blue oxidases and yellow oxidoreductases. Genes whose predicted proteins have secretory (Sec) pathway targeting signal sequences are in bold.

query	evalue	score	eggNOG_OGs	max_annot_lvl	Description	PFAMs
**TRINITY_DN735_c0_g1_i1.p1**	0.0	997.0	COG2272@1|root,KOG1516@2759|Eukaryota,3A34S@33154|Opisthokonta,3P2C3@4751|Fungi,3RMNF@4890|Ascomycota,20CU3@147545|Eurotiomycetes,3SA3I@5042|Eurotiales	4751|Fungi	Belongs to the type-B carboxylesterase lipase family	COesterase
**TRINITY_DN1075_c0_g1_i137.p2**	2.38e-158	452.0	COG3509@1|root,2SBTI@2759|Eukaryota,39SMX@33154|Opisthokonta,3NZNX@4751|Fungi,3QMGM@4890|Ascomycota,20HBI@147545|Eurotiomycetes,3S46R@5042|Eurotiales	4751|Fungi	Ferulic acid esterase (FaeA)	Esterase_phd
**TRINITY_DN641_c0_g1_i84.p6**	2.98e-114	339.0	KOG4569@1|root,KOG4569@2759|Eukaryota,3A0Y5@33154|Opisthokonta,3P2DI@4751|Fungi,3QTQB@4890|Ascomycota,20G1Q@147545|Eurotiomycetes,3S6C5@5042|Eurotiales	4751|Fungi	Lipase (class 3)	Lipase3_N,Lipase_3
TRINITY_DN980_c0_g1_i8.p1	3.93e-157	451.0	COG0657@1|root,KOG1515@2759|Eukaryota,3A11A@33154|Opisthokonta,3P291@4751|Fungi,3QUR6@4890|Ascomycota,20GUM@147545|Eurotiomycetes,3S3I8@5042|Eurotiales	4751|Fungi	Lipase esterase family protein	Abhydrolase_3
**TRINITY_DN28099_c0_g1_i1.p1**	0.0	971.0	KOG1325@1|root,KOG1325@2759|Eukaryota,38GBY@33154|Opisthokonta,3NUWK@4751|Fungi,3QJFV@4890|Ascomycota,20EF4@147545|Eurotiomycetes	4751|Fungi	Lysophospholipase	PLA2_B
TRINITY_DN1112_c0_g1_i19.p2	0.0	1254.0	COG1752@1|root,KOG2214@2759|Eukaryota,38RHC@33154|Opisthokonta,3NVQ8@4751|Fungi,3QPMP	4751|Fungi	Patatin-like phospholipase domain-containing protein	DUF3336,Patatin

			@4890|Ascomycota,20CJ0@147545|Eurotiomycetes,3S7TP@5042|Eurotiales			
TRINITY_DN624_c0_g1_i88.p1	0.0	1558.0	COG1502@1|root,KOG1329@2759|Eukaryota,38G6H@33154|Opisthokonta,3NX5P@4751|Fungi,3QJSM@4890|Ascomycota,20BUK@147545|Eurotiomycetes	4751|Fungi	phospholipase	PLDc,PLDc_2
TRINITY_DN6124_c0_g1_i1.p2	1.38e-109	326.0	COG2819@1|root,2SII4@2759|Eukaryota,3AKDR@33154|Opisthokonta,3PDMH@4751|Fungi,3RMNN@4890|Ascomycota,20U4J@147545|Eurotiomycetes	4751|Fungi	Putative esterase	Esterase
TRINITY_DN2326_c0_g1_i10.p1	0.0	1161.0	COG2132@1|root,COG3321@1|root,KOG1202@2759|Eukaryota,KOG1263@2759|Eukaryota,38D6P@33154|Opisthokonta,3NWH8@4751|Fungi,3QRMJ@4890|Ascomycota,20Z8D@147548|Leotiomycetes	4751|Fungi	Thioesterase domain	Acyl_transf_1,Ketoacyl-synt_C,PP-binding,PS-DH,SAT,Thioesterase,ketoacyl-synt
TRINITY_DN39836_c0_g1_i1.p1	7.27e-138	404.0	COG1893@1|root,2QPT5@2759|Eukaryota,39W61@33154|Opisthokonta,3NY0S@4751|Fungi,3QS3S@4890|Ascomycota,20C27@147545|Eurotiomycetes,3S4CP@5042|Eurotiales	4751|Fungi	2-dehydropantoate 2-reductase	ApbA,ApbA_C
TRINITY_DN1012_c0_g1_i20.p2	0.0	1964.0	KOG2408@1|root,KOG2408@2759|Eukaryota,38G3P@33154|Opisthokonta,3NWZ7@4751|Fungi,3QK9G@4890|Ascomycota,20DU3@147545|Eurotiomycetes,3S3SA@5042|Eurotiales	4751|Fungi	Bifunctional heme-containing enzyme that oxidizes linoleic acid to (8R,9Z,12Z)-8-hydroperoxyoctadeca-9,12-dienoate (within the N-terminal heme peroxidase domain)	An_peroxidase,p450
TRINITY_DN2502_c0_g1_i4.p1	4.44e-218	615.0	COG2124@1|root,KOG0156@2759|Eukaryota,38BSU@33154|Opisthokonta,3P0Q5@4751|Fungi,3QP8Z@4890|Ascomycota,20F8I@147545|Eurotiomycetes,3S7AW@5042|Eurotiales	4751|Fungi	cytochrome P450	p450
TRINITY_DN598_c0_g1_i180.p3	9.89e-194	558.0	COG2124@1|root,KOG0156@2759|Eukaryota,38BSU@33154|Opisthokonta,3P0Q5@4751|Fungi,3QP8Z@4890|Ascomycota,20F8I@147545|Eurotiomycetes	4751|Fungi	cytochrome P450	p450
TRINITY_DN177_c0_g2_i7.p7	2.93e-09	61.2	COG2124@1|root,KOG0157@2759|Eukaryota,38YJY@33154|Opisthokonta,3P14Z@4751|Fungi,3R0WT@4890|Ascomycota,21R68@147550|Sordariomycetes	4751|Fungi	Cytochrome p-450	p450
TRINITY_DN29817_c0_g1_i1.p1	0.0	970.0	COG1231@1|root,KOG0029@2759|Eukaryota,38B4W@33154|Opisthokonta,3P1AM@4751|Fungi,3R4J7@4890|Ascomycota,21Q1C@147550|Sordariomycetes,3TTQT@5125|Hypocreales,1FZ1N@110618|Nectriaceae	4751|Fungi	Endoribonuclease L-PSP	Amino_oxidase,Ribonuc_L-PSP

TRINITY_DN735_c0_g1_i1.p2	3.2e-231	653.0	COG3239@1|root,KOG4232@2759|Eukaryota,38B7V@33154|Opisthokonta,3NUM0@4751|Fungi,3QP5R@4890|Ascomycota,212T1@147550|Sordariomycetes	4751|Fungi	Fatty acid desaturase	Cyt-b5,FA_desaturase
**TRINITY_DN36397_c0_g1_i1.p1**	1.12e-155	453.0	2A2M4@1|root,2RY3D@2759|Eukaryota,39Z1J@33154|Opisthokonta,3NWH4@4751|Fungi,3R6GX@4890|Ascomycota,20T2M@147545|Eurotiomycetes,3SFDW@5042|Eurotiales	4751|Fungi	Glycosyl hydrolase family 61	CBM_1,Glyco_hydro_61
TRINITY_DN988_c0_g1_i2.p3	2.74e-279	777.0	KOG2132@1|root,KOG2132@2759|Eukaryota,38CB5@33154|Opisthokonta,3NZH4@4751|Fungi,3QN14@4890|Ascomycota,20E5C@147545|Eurotiomycetes,3S86W@5042|Eurotiales	4751|Fungi	JmjC domain protein	Cupin_8
**TRINITY_DN31824_c0_g1_i1.p1**	0.0	883.0	COG2132@1|root,KOG1263@2759|Eukaryota,39UM9@33154|Opisthokonta,3NVU0@4751|Fungi,3QJPD@4890|Ascomycota,20BGD@147545|Eurotiomycetes,3SAXV@5042|Eurotiales	4751|Fungi	multicopper oxidase	Cu-oxidase,Cu-oxidase_2,Cu-oxidase_3
TRINITY_DN39606_c0_g1_i1.p2	5.2e-208	583.0	COG0404@1|root,KOG2844@2759|Eukaryota,39WG2@33154|Opisthokonta,3P08Y@4751|Fungi,3QQDP@4890|Ascomycota,20SJP@147545|Eurotiomycetes	4751|Fungi	N,N-dimethylglycine oxidase	DAO
TRINITY_DN594_c0_g1_i7.p3	2.96e-84	276.0	COG0654@1|root,KOG3855@2759|Eukaryota,39RF9@33154|Opisthokonta,3NWEM@4751|Fungi,3QKPM@4890|Ascomycota,217NA@147550|Sordariomycetes,3THR1@5125|Hypocreales	4751|Fungi	Phenol 2-monooxygenase	FAD_binding_3,Phe_hydrox_dim
TRINITY_DN594_c0_g1_i7.p4	5.05e-29	123.0	COG0654@1|root,KOG3855@2759|Eukaryota,39RF9@33154|Opisthokonta,3NWEM@4751|Fungi,3QKPM@4890|Ascomycota,217NA@147550|Sordariomycetes,3THR1@5125|Hypocreales	4751|Fungi	Phenol 2-monooxygenase	FAD_binding_3,Phe_hydrox_dim
TRINITY_DN624_c0_g1_i88.p6	2.89e-117	357.0	COG0654@1|root,KOG3855@2759|Eukaryota,39RF9@33154|Opisthokonta,3NWEM@4751|Fungi,3QKPM@4890|Ascomycota,20BCX@147545|Eurotiomycetes,3S5CI@5042|Eurotiales	4751|Fungi	Phenol hydroxylase, C-terminal dimerisation domain	FAD_binding_3,Phe_hydrox_dim
TRINITY_DN624_c0_g1_i88.p4	1.07e-193	557.0	COG0654@1|root,KOG3855@2759|Eukaryota,39RF9@33154|Opisthokonta,3NWEM@4751|Fungi,3QKPM@4890|Ascomycota,20BCX@147545|Eurotiomycetes,3S5CI@5042|Eurotiales	4751|Fungi	Phenol hydroxylase, C-terminal dimerisation domain	FAD_binding_3,Phe_hydrox_dim
TRINITY_DN360_c0_g1_i9.p2	4.77e-160	456.0	KOG1591@1|root,KOG1591@2759|Eukaryota,39TQ9@33154|Opisthokonta,3P14U@4751|Fungi,3QTUR@4890|Ascomycota,20GQA@147545|Eurotiomycetes,3SEM1@5042|Eurotiales	4751|Fungi	Prolyl 4-hydroxylase alpha subunit homologues.	2OG-FeII_Oxy_3

TRINITY_DN52_c0_g1_i130.p6	4.54e-173	484.0	COG3467@1|root,2RYSK@2759|Eukaryota,39K0W@33154|Opisthokonta,3Q4DP@4751|Fungi,3RMK0@4890|Ascomycota,20U1M@147545|Eurotiomycetes,3S85F@5042|Eurotiales	4751|Fungi	Pyridoxamine 5'-phosphate oxidase	Pyridox_ox_2
TRINITY_DN1198_c0_g1_i2.p1	5.15e-312	866.0	COG2303@1|root,KOG1238@2759|Eukaryota,38UEK@33154|Opisthokonta,3NWDN@4751|Fungi,3QJNB@4890|Ascomycota,216R9@147550|Sordariomycetes	4751|Fungi	Belongs to the GMC oxidoreductase family	GMC_oxred_C,GMC_oxred_N
**TRINITY_DN2698_c0_g1_i1.p2**	1.08e-102	315.0	COG0277@1|root,2SJ3M@2759|Eukaryota,38E49@33154|Opisthokonta,3NZB0@4751|Fungi,3QMRQ@4890|Ascomycota,20EPT@147545|Eurotiomycetes,3SBA3@5042|Eurotiales	4751|Fungi	Belongs to the oxygen-dependent FAD-linked oxidoreductase family	BBE,FAD_binding_4
TRINITY_DN2698_c0_g1_i1.p3	1.63e-62	207.0	COG0277@1|root,2SJ3M@2759|Eukaryota,38E49@33154|Opisthokonta,3NZB0@4751|Fungi,3QMRQ@4890|Ascomycota,20EPT@147545|Eurotiomycetes,3SBA3@5042|Eurotiales	4751|Fungi	Belongs to the oxygen-dependent FAD-linked oxidoreductase family	BBE,FAD_binding_4
TRINITY_DN1164_c0_g1_i4.p1	1.07e-178	510.0	COG0665@1|root,KOG2820@2759|Eukaryota,39SVA@33154|Opisthokonta,3NVW5@4751|Fungi,3QRW0@4890|Ascomycota,20JXR@147545|Eurotiomycetes	4751|Fungi	FAD dependent oxidoreductase	DAO
TRINITY_DN29566_c0_g1_i1.p1	5.03e-141	409.0	COG0665@1|root,KOG3923@2759|Eukaryota,3A3CA@33154|Opisthokonta,3P3MG@4751|Fungi,3QU5Z@4890|Ascomycota,21ACN@147550|Sordariomycetes,3TWEY@5125|Hypocreales,1FWW0@110618|Nectriaceae	4751|Fungi	FAD dependent oxidoreductase	DAO
TRINITY_DN40225_c0_g1_i4.p2	2.08e-192	550.0	COG0665@1|root,2QVCU@2759|Eukaryota,38FM7@33154|Opisthokonta,3NUEI@4751|Fungi,3R2WX@4890|Ascomycota,218AD@147550|Sordariomycetes,1EV3K@1028384|Glomerellales	4751|Fungi	FAD dependent oxidoreductase	DAO
TRINITY_DN6667_c0_g1_i1.p1	1.12e-140	418.0	2CWIY@1|root,2RUPG@2759|Eukaryota,39UEZ@33154|Opisthokonta,3P045@4751|Fungi,3QQT5@4890|Ascomycota,2000C@147541|Dothideomycetes	4751|Fungi	FAD dependent oxidoreductase	DAO
TRINITY_DN572_c1_g1_i171.p5	1.49e-231	646.0	COG1902@1|root,KOG0134@2759|Eukaryota,39RM1@33154|Opisthokonta,3NUUE@4751|@147545|Eurotiomycetes	4751|Fungi	flavin oxidoreductase	Oxidored_FMN
TRINITY_DN1964_c0_g1_i1.p2	1.41e-152	450.0	COG2303@1|root,KOG1238@2759|Eukaryota,38UEK@33154|Opisthokonta,3NWDN@4751|Fungi,3QJNB@4890|Ascomycota,20BXA@147545|Eurotiomycetes,3SABT@5042|Eurotiales	4751|Fungi	GMC oxidoreductase	GMC_oxred_C,GMC_oxred_N

TRINITY_DN11_c0_g2_i1.p1	7.51e-130	382.0	COG2130@1|root,KOG1196@2759|Eukaryota,39AU4@33154|Opisthokonta,3NWMF@4751|Fungi,3QPK5@4890|Ascomycota,20DHF@147545|Eurotiomycetes	4751|Fungi	N-terminal domain of oxidoreductase	ADH_N_2,ADH_zinc_N
TRINITY_DN572_c1_g1_i171.p6	6.2e-226	625.0	COG2070@1|root,2QTWN@2759|Eukaryota,397Z1@33154|Opisthokonta,3NWW5@4751|Fungi,3QKGZ@4890|Ascomycota,20E39@147545|Eurotiomycetes,3S2ZP@5042|Eurotiales	4751|Fungi	Oxidoreductase, 2-nitropropane dioxygenase family	NMO
TRINITY_DN4608_c0_g1_i6.p3	1.64e-157	459.0	COG1252@1|root,KOG2495@2759|Eukaryota,3A0NB@33154|Opisthokonta,3P11E@4751|Fungi,3QR9P@4890|Ascomycota,20DKU@147545|Eurotiomycetes	4751|Fungi	Pyridine nucleotide-disulphide oxidoreductase	Pyr_redox_2
TRINITY_DN31673_c0_g1_i1.p1	1.57e-198	556.0	COG0604@1|root,KOG1198@2759|Eukaryota,38G12@33154|Opisthokonta,3NX3C@4751|Fungi,3QMAW@4890|Ascomycota,20AQ8@147545|Eurotiomycetes,3S79S@5042|Eurotiales	4751|Fungi	Zinc-binding oxidoreductase	ADH_N,ADH_zinc_N
TRINITY_DN3014_c0_g1_i1.p3	4.98e-270	743.0	COG2220@1|root,KOG3798@2759|Eukaryota,39UP3@33154|Opisthokonta,3NX7Q@4751|Fungi,3QQ6X@4890|Ascomycota,20CK9@147545|Eurotiomycetes,3S6JU@5042|Eurotiales	4751|Fungi	Zn-dependent hydrolase oxidoreductase family protein	Lactamase_B_2

Table S7: Results of differentially expressed genes analysis of the fungus *Aspergillus puulaauensis* (EXF-7678) grown on synthetic material Lascaux 498 obtained using the EggNOG online database. Genes for esterases and oxidases with reduced expression levels are shown. The orange color represents the group of esterases, blue oxidase and yellow oxidoreductase. Genes whose predicted proteins have secretory (Sec) pathway targeting signal sequences are in bold.

query	evalue	score	eggNOG_OGs	max_annot_lvl	Description	PFAMs
TRINITY_DN1019_c1_g1_i21.p5	1.82e-71	224.0	COG1946@1|root,KOG3016@2759|Eukaryota,38GG7@33154|Opisthokonta,3NXST@4751|Fungi,3QP0K@4890|Ascomycota,20FHE@147545|Eurotiomycetes,3S67V@5042|Eurotiales	4751|Fungi	acyl-CoA thioesterase II	4HBT_3,Acyl_CoA_thio
TRINITY_DN1019_c1_g1_i21.p2	1.71e-99	298.0	COG1946@1|root,KOG3016@2759|Eukaryota,38GG7@33154|Opisthokonta,3NXST@4751|Fungi,3QP0K@4890|Ascomycota,20FHE@147545|Eurotiomycetes,3S67V@5042|Eurotiales	4751|Fungi	acyl-CoA thioesterase II	4HBT_3,Acyl_CoA_thio
**TRINITY_DN1788_c0_g1_i1.p1**	7.47e-153	452.0	2EB3Y@1|root,2SH94@2759|Eukaryota,39VZP@33154|Opisthokonta,3NZDK@4751|Fungi,3QK4D	4751|Fungi	Belongs to the tannase family	Tannase

			@4890|Ascomycota,20BI2@147545|Eurotiomycetes			
**TRINITY_DN10834_c0_g1_i2.p6**	8.95e-31	118.0	COG2272@1|root,KOG4389@2759|Eukaryota,38BW2@33154|Opisthokonta,3NWXI@4751|Fungi,3QRM0@4890|Ascomycota,20GHB@147545|Eurotiomycetes,3S75I@5042|Eurotiales	4751|Fungi	Belongs to the type-B carboxylesterase lipase family	COesterase,TB2_DP1_HVA22
TRINITY_DN10834_c0_g1_i2.p1	4.43e-240	672.0	COG2272@1|root,KOG4389@2759|Eukaryota,38BW2@33154|Opisthokonta,3NWXI@4751|Fungi,3QRM0@4890|Ascomycota,20GHB@147545|Eurotiomycetes,3S75I@5042|Eurotiales	4751|Fungi	Belongs to the type-B carboxylesterase lipase family	COesterase,TB2_DP1_HVA22
TRINITY_DN123_c0_g1_i7.p2	0.0	929.0	COG2272@1|root,KOG1516@2759|Eukaryota,38EQ2@33154|Opisthokonta,3NX7C@4751|Fungi,3QR31@4890|Ascomycota,20H1F@147545|Eurotiomycetes	4751|Fungi	Belongs to the type-B carboxylesterase lipase family	COesterase,DnaJ,zf-CSL
**TRINITY_DN2865_c0_g1_i1.p2**	2.86e-29	119.0	COG2272@1|root,KOG1516@2759|Eukaryota,38D2A@33154|Opisthokonta,3PCVC@4751|Fungi,3RN9G@4890|Ascomycota,21RNJ@147550|Sordariomycetes,1F1VQ@1028384|Glomerellales	4751|Fungi	Belongs to the type-B carboxylesterase lipase family	COesterase
TRINITY_DN2865_c0_g1_i1.p1	9.18e-164	478.0	COG2272@1|root,KOG4389@2759|Eukaryota,3AG57@33154|Opisthokonta,3PCYD@4751|Fungi,3R0Y0@4890|Ascomycota,204EC@147541|Dothideomycetes,3MN4R@451867|Dothideomycetidae	4751|Fungi	Belongs to the type-B carboxylesterase lipase family	COesterase
TRINITY_DN353_c0_g1_i127.p2	8.59e-61	208.0	COG2272@1|root,KOG4389@2759|Eukaryota,397KR@33154|Opisthokonta,3P02N@4751|Fungi,3QP6C@4890|Ascomycota,20T70@147545|Eurotiomycetes,3SFGB@5042|Eurotiales	4751|Fungi	Belongs to the type-B carboxylesterase lipase family	COesterase
**TRINITY_DN353_c0_g1_i151.p1**	4.85e-167	491.0	COG2272@1|root,KOG4389@2759|Eukaryota,397KR@33154|Opisthokonta,3P02N@4751|Fungi,3QP6C@4890|Ascomycota,20T70@147545|Eurotiomycetes,3SFGB@5042|Eurotiales	4751|Fungi	Belongs to the type-B carboxylesterase lipase family	COesterase
**TRINITY_DN831_c0_g1_i1.p2**	1.55e-94	281.0	2A1QU@1|root,2SI38@2759|Eukaryota,3A01F@33154|Opisthokonta,3P2ER@4751|Fungi,3QTNS@4890|Ascomycota	4751|Fungi	Catalyzes the hydrolysis of cutin, a polyester that forms the structure of plant cuticle	Cutinase
TRINITY_DN10834_c0_g1_i2.p3	2.32e-81	248.0	2CNAV@1|root,2QUVW@2759|Eukaryota,38RB9@33154|Opisthokonta,3NZ4Y@4751|Fungi,3QT8M@4890|Ascomycota,20KG2@147545|Eurotiomycetes,3S400@5042|Eurotiales	4751|Fungi	GDSL-like lipase acylhydrolase domain protein	Lipase_GDSL,Lipase_GDSL_2

**TRINITY_DN1106_c0_g1_i1.p2**	1.35e-142	411.0	KOG4569@1|root,KOG4569@2759|Eukaryota,3A0Y5@33154|Opisthokonta,3P2DI@4751|Fungi,3QTQB@4890|Ascomycota,20G1Q@147545|Eurotiomycetes	4751|Fungi	Lipase (class 3)	Lipase3_N,Lipase_3
**TRINITY_DN40286_c1_g1_i1.p1**	8.38e-101	303.0	KOG4569@1|root,KOG4569@2759|Eukaryota,3A0Y5@33154|Opisthokonta,3P3PN@4751|Fungi,3QVPQ@4890|Ascomycota,21F1I@147550|Sordariomycetes	4751|Fungi	Lipase (class 3)	Lipase_3
**TRINITY_DN716_c1_g1_i1.p2**	8.86e-181	520.0	2C2TR@1|root,2QSW8@2759|Eukaryota,38F0U@33154|Opisthokonta,3NZ2A@4751|Fungi,3QQ18@4890|Ascomycota,20GJ1@147545|Eurotiomycetes,3S6EU@5042|Eurotiales	4751|Fungi	Lipase b	-
**TRINITY_DN716_c1_g1_i10.p2**	8.86e-181	520.0	2C2TR@1|root,2QSW8@2759|Eukaryota,38F0U@33154|Opisthokonta,3NZ2A@4751|Fungi,3QQ18@4890|Ascomycota,20GJ1@147545|Eurotiomycetes,3S6EU@5042|Eurotiales	4751|Fungi	Lipase b	-
**TRINITY_DN2204_c0_g1_i22.p1**	0.0	997.0	KOG1325@1|root,KOG1325@2759|Eukaryota,38GBY@33154|Opisthokonta,3NUWK@4751|Fungi,3QJFV@4890|Ascomycota,20EF4@147545|Eurotiomycetes,3S5E2@5042|Eurotiales	4751|Fungi	Lysophospholipase	PLA2_B
TRINITY_DN1374_c0_g1_i1.p4	1.86e-68	218.0	28PJD@1|root,2QW7I@2759|Eukaryota,39TJA@33154|Opisthokonta,3NWYV@4751|Fungi,3QM7J@4890|Ascomycota,20PGF@147545|Eurotiomycetes,3S7FW@5042|Eurotiales	4751|Fungi	Pectinolytic enzyme	-
TRINITY_DN1374_c0_g1_i1.p3	5.18e-63	205.0	28PJD@1|root,2QW7I@2759|Eukaryota,39TJA@33154|Opisthokonta,3NWYV@4751|Fungi,3QM7J@4890|Ascomycota,20PGF@147545|Eurotiomycetes,3S7FW@5042|Eurotiales	4751|Fungi	Pectinolytic enzyme	-
TRINITY_DN7272_c0_g1_i24.p1	0.0	3570.0	COG3321@1|root,KOG1202@2759|Eukaryota,38D6P@33154|Opisthokonta,3NWH8@4751|Fungi,3QRMJ@4890|Ascomycota,20CQP@147545|Eurotiomycetes,3S57S@5042|Eurotiales	4751|Fungi	polyketide synthase	Acyl_transf_1,KAsynt_C_assoc,Ketoacyl-synt_C,PSDH,SAT,Thioesterase,ketoacyl-synt
**TRINITY_DN23264_c0_g1_i1.p1**	4.14e-53	192.0	2ECT2@1|root,2SIJX@2759|Eukaryota,3AF6W@33154|Opisthokonta,3PAS4@4751|Fungi,3QP10@4890|Ascomycota,21NW3@147550|Sordariomycetes,3UEQ3@5139|Sordariales,3HFQH@35718|Chaetomiaceae	4751|Fungi	Repeat domain in Vibrio, Colwellia, Bradyrhizobium and Shewanella	Lipase_GDSL_2,VCBS
**TRINITY_DN277_c0_g4_i4.p1**	2.94e-163	472.0	2CHYE@1|root,2QUCT@2759|Eukaryota,38VB8@33154|Opisthokonta,3NZ4K@4751|Fungi,3QT35	4751|Fungi	Serum paraoxonase arylesterase family protein	Arylesterase

			@4890|Ascomycota,20KCQ@147545|Eurotiomycetes,3S4RM@5042|Eurotiales			
TRINITY_DN6134_c0_g1_i58.p4	1.34e-149	429.0	2ER90@1|root,2SU3R@2759|Eukaryota,3AVNA@33154|Opisthokonta,3PHZF@4751|Fungi,3R5FK@4890|Ascomycota,20NP4@147545|Eurotiomycetes,3S9K6@5042|Eurotiales	4751|Fungi	Thioesterase-like superfamily	4HBT_3
TRINITY_DN6134_c0_g1_i70.p4	1.34e-149	429.0	2ER90@1|root,2SU3R@2759|Eukaryota,3AVNA@33154|Opisthokonta,3PHZF@4751|Fungi,3R5FK@4890|Ascomycota,20NP4@147545|Eurotiomycetes,3S9K6@5042|Eurotiales	4751|Fungi	Thioesterase-like superfamily	4HBT_3
TRINITY_DN400_c0_g1_i88.p7	4.05e-227	629.0	28JWX@1|root,2QSB5@2759|Eukaryota,38J5S@33154|Opisthokonta,3NU2X@4751|Fungi,3QK31@4890|Ascomycota,20BIA@147545|Eurotiomycetes,3S6P5@5042|Eurotiales	4751|Fungi	alternative oxidase	AOX
TRINITY_DN400_c0_g1_i88.p8	4.05e-227	629.0	28JWX@1|root,2QSB5@2759|Eukaryota,38J5S@33154|Opisthokonta,3NU2X@4751|Fungi,3QK31@4890|Ascomycota,20BIA@147545|Eurotiomycetes,3S6P5@5042|Eurotiales	4751|Fungi	alternative oxidase	AOX
**TRINITY_DN2003_c0_g1_i6.p6**	2.31e-47	165.0	COG2132@1|root,KOG1263@2759|Eukaryota,3AJ0P@33154|Opisthokonta,3PA2X@4751|Fungi,3QT7B@4890|Ascomycota,20DN4@147545|Eurotiomycetes,3S722@5042|Eurotiales	4751|Fungi	Belongs to the multicopper oxidase family	Cu-oxidase,Cu-oxidase_2,Cu-oxidase_3
TRINITY_DN2003_c0_g1_i6.p1	3.48e-213	608.0	COG2132@1|root,KOG1263@2759|Eukaryota,3AJ0P@33154|Opisthokonta,3PA2X@4751|Fungi,3QT7B@4890|Ascomycota,20DN4@147545|Eurotiomycetes,3S722@5042|Eurotiales	4751|Fungi	Belongs to the multicopper oxidase family	Cu-oxidase,Cu-oxidase_2,Cu-oxidase_3
**TRINITY_DN3517_c0_g1_i1.p1**	3.07e-297	825.0	COG2132@1|root,KOG1263@2759|Eukaryota,39Y4Z@33154|Opisthokonta,3NZUY@4751|Fungi,3RJIS@4890|Ascomycota,20T69@147545|Eurotiomycetes,3S42R@5042|Eurotiales	4751|Fungi	Belongs to the multicopper oxidase family	Cu-oxidase,Cu-oxidase_2,Cu-oxidase_3
TRINITY_DN1799_c0_g1_i4.p1	2.98e-130	374.0	COG3000@1|root,KOG0873@2759|Eukaryota,38C6I@33154|Opisthokonta,3NU50@4751|Fungi,3QK2J@4890|Ascomycota,20AVK@147545|Eurotiomycetes,3S7FH@5042|Eurotiales	4751|Fungi	Belongs to the sterol desaturase family	FA_hydroxylase
TRINITY_DN230_c0_g1_i6.p6	7.14e-104	307.0	COG3000@1|root,KOG0873@2759|Eukaryota,38C6I@33154|Opisthokonta,3NU50@4751|Fungi,3QK2J@4890|Ascomycota,20AVK@147545|Eurotiomycetes	4751|Fungi	Belongs to the sterol desaturase family	FA_hydroxylase

TRINITY_DN51_c0_g4_i1.p2	1.47e-154	458.0	COG2124@1|root,KOG0156@2759|Eukaryota,38BSU@33154|Opisthokonta,3NUFV@4751|Fungi,3QSVE@4890|Ascomycota,20FN2@147545|Eurotiomycetes,3SCJJ@5042|Eurotiales	4751|Fungi	Catalyzes the conversion of tryprostatin A to fumitremorgin C	p450
TRINITY_DN867_c0_g1_i8.p9	7.82e-41	138.0	KOG3469@1|root,KOG3469@2759|Eukaryota,3A3WN@33154|Opisthokonta,3P7D2@4751|Fungi,3RJSZ@4890|Ascomycota,20TBC@147545|Eurotiomycetes,3SFI8@5042|Eurotiales	4751|Fungi	Cytochrome c oxidase subunit 6A, mitochondrial	COX6A
TRINITY_DN598_c0_g1_i253.p3	9.89e-194	558.0	COG2124@1|root,KOG0156@2759|Eukaryota,38BSU@33154|Opisthokonta,3P0Q5@4751|Fungi,3QP8Z@4890|Ascomycota,20F8I@147545|Eurotiomycetes	4751|Fungi	cytochrome P450	p450
TRINITY_DN598_c0_g1_i297.p2	3.54e-160	469.0	COG2124@1|root,KOG0156@2759|Eukaryota,38BSU@33154|Opisthokonta,3P0Q5@4751|Fungi,3QP8Z@4890|Ascomycota,20F8I@147545|Eurotiomycetes	4751|Fungi	cytochrome P450	p450
TRINITY_DN612_c0_g1_i11.p10	5.45e-66	204.0	COG3474@1|root,KOG3453@2759|Eukaryota,3A3GP@33154|Opisthokonta,3P3WW@4751|Fungi,3QW2P@4890|Ascomycota,20HWP@147545|Eurotiomycetes,3SD0S@5042|Eurotiales	4751|Fungi	Electron carrier protein	Cytochrom_C
TRINITY_DN1477_c0_g1_i5.p1	1.77e-79	251.0	COG0654@1|root,KOG2614@2759|Eukaryota,39VWK@33154|Opisthokonta,3NXRU@4751|Fungi,3QMHA@4890|Ascomycota,20SJV@147545|Eurotiomycetes,3SF87@5042|Eurotiales	4751|Fungi	FAD binding domain	FAD_binding_2,FAD_binding_3
TRINITY_DN1477_c0_g1_i5.p2	4.59e-99	300.0	COG0654@1|root,KOG2614@2759|Eukaryota,39VWK@33154|Opisthokonta,3NXRU@4751|Fungi,3QMHA@4890|Ascomycota,20SJV@147545|Eurotiomycetes,3SF87@5042|Eurotiales	4751|Fungi	FAD binding domain	FAD_binding_2,FAD_binding_3
TRINITY_DN599_c0_g1_i4.p3	2.1e-40	145.0	COG0654@1|root,KOG2614@2759|Eukaryota,39U2S@33154|Opisthokonta,3P1EC@4751|Fungi,3QTMT@4890|Ascomycota,20F6Y@147545|Eurotiomycetes,3S93H@5042|Eurotiales	4751|Fungi	FAD binding domain	FAD_binding_3
TRINITY_DN599_c0_g1_i4.p2	1.21e-30	120.0	COG0654@1|root,KOG2614@2759|Eukaryota,39U2S@33154|Opisthokonta,3P1EC@4751|Fungi,3QTMT@4890|Ascomycota,20F6Y@147545|Eurotiomycetes,3S93H@5042|Eurotiales	4751|Fungi	FAD binding domain	FAD_binding_3
TRINITY_DN1288_c0_g1_i3.p1	5.52e-306	845.0	COG0536@1|root,KOG1489@2759|Eukaryota,38B68@33154|Opisthokonta,3NXIX@4751|Fungi,3Q	4751|Fungi	GTP-binding protein	GTP1_OBG,MMR_HSR1

			NT8@4890|Ascomycota,20AU0@147545|Eurotiomycetes,3S6J9@5042|Eurotiales			
TRINITY_DN3586_c0_g1_i4.p6	2.1e-39	146.0	2EE88@1|root,2SJPM@2759|Eukaryota,38XCS@33154|Opisthokonta,3PP8R@4751|Fungi,3R8HY@4890|Ascomycota,21K2U@147550|Sordariomycetes,3UEG7@5139|Sordariales,3HGBW@35718|Chaetomiaceae	4751|Fungi	Luciferase-like monooxygenase	Bac_luciferase
TRINITY_DN2979_c0_g1_i3.p2	0.0	924.0	COG2072@1|root,KOG1399@2759|Eukaryota,38D94@33154|Opisthokonta,3NZ5U@4751|Fungi,3QK71@4890|Ascomycota,20AYG@147545|Eurotiomycetes	4751|Fungi	monooxygenase	FMO-like,K_oxygenase,NAD_binding_8,Pyr_redox_2
**TRINITY_DN1569_c0_g1_i2.p1**	2.02e-98	296.0	28MC9@1|root,2QTVQ@2759|Eukaryota,38FAF@33154|Opisthokonta,3P0PR@4751|Fungi,3QS2U@4890|Ascomycota,20SJZ@147545|Eurotiomycetes,3S9DW@5042|Eurotiales	4751|Fungi	Peroxidase, family 2	Peroxidase_2
**TRINITY_DN1944_c0_g1_i1.p2**	4.82e-171	488.0	2C2B4@1|root,2S6CG@2759|Eukaryota,38FWW@33154|Opisthokonta,3NZAE@4751|Fungi,3QJYE@4890|Ascomycota,20ECW@147545|Eurotiomycetes,3S8AC@5042|Eurotiales	4751|Fungi	Peroxidase, family 2	Peroxidase_2
**TRINITY_DN1944_c0_g1_i1.p5**	2.69e-28	114.0	2C2B4@1|root,2S6CG@2759|Eukaryota,38FWW@33154|Opisthokonta,3NZAE@4751|Fungi,3QJYE@4890|Ascomycota,20ECW@147545|Eurotiomycetes,3S8AC@5042|Eurotiales	4751|Fungi	Peroxidase, family 2	Peroxidase_2
TRINITY_DN23211_c0_g1_i1.p1	1.97e-169	482.0	2E612@1|root,2SCSP@2759|Eukaryota,39TM5@33154|Opisthokonta,3PQ12@4751|Fungi,3R9EG@4890|Ascomycota,21KCS@147550|Sordariomycetes,1F5NI@1028384|Glomerellales	4751|Fungi	Phytanoyl-CoA dioxygenase (PhyH)	PhyH
TRINITY_DN2239_c0_g1_i6.p1	6.9e-273	761.0	COG1231@1|root,KOG0029@2759|Eukaryota,38F9V@33154|Opisthokonta,3NTXA@4751|Fungi,3QKSW@4890|Ascomycota,20EFU@147545|Eurotiomycetes,3S854@5042|Eurotiales	4751|Fungi	Polyamine oxidase	Amino_oxidase
TRINITY_DN5929_c0_g1_i21.p4	3.62e-241	667.0	COG0259@1|root,KOG2586@2759|Eukaryota,38HH9@33154|Opisthokonta,3NZQH@4751|Fungi,3QJCE@4890|Ascomycota,20BQE@147545|Eurotiomycetes	4751|Fungi	Pyridoxamine 5'-phosphate oxidase	PNP_phzG_C,Putative_PNPOx
TRINITY_DN2279_c0_g1_i2.p2	1.09e-49	159.0	KOG4148@1|root,2S8TH@2759|Eukaryota,3ABWX@33154|Opisthokonta,3Q3CK@4751|Fungi,3RKE2@4890|Ascomycota,20TNC@147545|Eurotiomycetes,3S9M3@5042|Eurotiales	4751|Fungi	Required for mitochondrial cytochrome c oxidase (COX) assembly and respiration	Cmc1

**TRINITY_DN7522_c0_g1_i1.p1**	4.53e-267	745.0	COG0520@1|root,KOG2142@2759|Eukaryota,38F30@33154|Opisthokonta,3NXCU@4751|Fungi,3QPCH@4890|Ascomycota,20BB9@147545|Eurotiomycetes,3S7CK@5042|Eurotiales	4751|Fungi	Sulfurates the molybdenum cofactor.	Aminotran_5,MOSC,MOSC_N
**TRINITY_DN27860_c0_g1_i1.p1**	1.97e-242	670.0	29DZU@1|root,2RM4B@2759|Eukaryota,39XMX@33154|Opisthokonta,3NX6H@4751|Fungi,3QRNN@4890|Ascomycota,20RSW@147545|Eurotiomycetes,3SEY6@5042|Eurotiales	4751|Fungi	tyrosinase	Tyrosinase
**TRINITY_DN1032_c0_g1_i3.p2**	1.91e-71	233.0	COG2303@1|root,KOG1238@2759|Eukaryota,38UEK@33154|Opisthokonta,3NUFD@4751|Fungi,3QKEH@4890|Ascomycota,20DI7@147545|Eurotiomycetes	4751|Fungi	Belongs to the GMC oxidoreductase family	GMC_oxred_C,GMC_oxred_N
TRINITY_DN31588_c2_g1_i2.p1	7.62e-306	844.0	COG2303@1|root,KOG1238@2759|Eukaryota,38GFW@33154|Opisthokonta,3NVTR@4751|Fungi,3QPVA@4890|Ascomycota,20AQP@147545|Eurotiomycetes,3SA1S@5042|Eurotiales	4751|Fungi	Belongs to the GMC oxidoreductase family	GMC_oxred_C,GMC_oxred_N
TRINITY_DN74_c0_g1_i18.p1	4.81e-254	712.0	COG2303@1|root,KOG1238@2759|Eukaryota,39MV1@33154|Opisthokonta,3NYBM@4751|Fungi,3QK2K@4890|Ascomycota,20D4Q@147545|Eurotiomycetes,3S3K1@5042|Eurotiales	4751|Fungi	Belongs to the GMC oxidoreductase family	GMC_oxred_C,GMC_oxred_N
TRINITY_DN51_c0_g4_i1.p3	4.25e-44	156.0	COG3491@1|root,COG3491@2|Bacteria,1MUNT@1224|Proteobacteria,1RSQ2@1236|Gammaproteobacteria,1Z1SJ@136846|Pseudomonas stutzeri group	1236|Gammaproteobacteria	Belongs to the iron ascorbate-dependent oxidoreductase family	2OG-FeII_Oxy,DIOX_N
TRINITY_DN2168_c0_g1_i3.p2	4.36e-82	260.0	28PNZ@1|root,2QWB6@2759|Eukaryota,39SEF@33154|Opisthokonta,3P15H@4751|Fungi,3QSEX@4890|Ascomycota,20FEG@147545|Eurotiomycetes,3S3Q6@5042|Eurotiales	4751|Fungi	Belongs to the oxygen-dependent FAD-linked oxidoreductase family	BBE,FAD_binding_4
**TRINITY_DN2168_c0_g1_i3.p1**	1.11e-103	318.0	28PNZ@1|root,2QWB6@2759|Eukaryota,39SEF@33154|Opisthokonta,3P15H@4751|Fungi,3QSEX@4890|Ascomycota,20FEG@147545|Eurotiomycetes,3S3Q6@5042|Eurotiales	4751|Fungi	Belongs to the oxygen-dependent FAD-linked oxidoreductase family	BBE,FAD_binding_4
TRINITY_DN440_c0_g1_i152.p2	5.69e-179	510.0	COG0277@1|root,2S2FH@2759|Eukaryota,39K11@33154|Opisthokonta,3NZSZ@4751|Fungi,3RMK6@4890|Ascomycota,20U1V@147545|Eurotiomycetes,3SE1A@5042|Eurotiales	4751|Fungi	Belongs to the oxygen-dependent FAD-linked oxidoreductase family	BBE,FAD_binding_4
**TRINITY_DN7484_c0_g1_i1.p1**	3.22e-248	705.0	COG0277@1|root,2QUV4@2759|Eukaryota,38Z80@33154|Opisthokonta,3NXMG@4751|Fungi,3QNI	4751|Fungi	Belongs to the oxygen-dependent FAD-linked oxidoreductase family	BBE,FAD_binding_4

			M@4890|Ascomycota,2042J@147541|Dothideomycetes			
TRINITY_DN2231_c0_g1_i1.p2	1.02e-111	331.0	COG0665@1|root,KOG3923@2759|Eukaryota,3AEPJ@33154|Opisthokonta,3P2G9@4751|Fungi,3QU3X@4890|Ascomycota,20S3Z@147545|Eurotiomycetes,3S4NH@5042|Eurotiales	4751|Fungi	FAD dependent oxidoreductase	DAO
TRINITY_DN395_c0_g1_i2.p2	7.2e-48	163.0	COG0665@1|root,KOG3923@2759|Eukaryota,39SQP@33154|Opisthokonta,3NZYG@4751|Fungi,3QR57@4890|Ascomycota,20ZAN@147548|Leotiomycetes	4751|Fungi	FAD dependent oxidoreductase	DAO
TRINITY_DN395_c0_g1_i2.p1	1.39e-71	224.0	COG0665@1|root,KOG3923@2759|Eukaryota,39SQP@33154|Opisthokonta,3NZYG@4751|Fungi,3QR57@4890|Ascomycota,21FJ1@147550|Sordariomycetes,3TPMS@5125|Hypocreales,1FWJY@110618|Nectriaceae	4751|Fungi	FAD dependent oxidoreductase	DAO
TRINITY_DN50_c1_g1_i2.p1	2.05e-10	63.9	COG2303@1|root,KOG1238@2759|Eukaryota,38UEK@33154|Opisthokonta,3NXH9@4751|Fungi,3QQ5Z@4890|Ascomycota,212HC@147550|Sordariomycetes,41QIA@639021|Magnaporthales	4751|Fungi	GMC oxidoreductase	GMC_oxred_C,GMC_oxred_N
TRINITY_DN488_c0_g2_i3.p1	1.91e-270	745.0	COG2084@1|root,KOG0409@2759|Eukaryota,38HZR@33154|Opisthokonta,3P012@4751|Fungi,3QM7M@4890|Ascomycota,20E2D@147545|Eurotiomycetes,3S45S@5042|Eurotiales	4751|Fungi	Oxidoreductase, acting on the CH-OH group of donors, NAD or NADP as acceptor	NAD_binding_11,NAD_binding_2,Pre-SET,SET
TRINITY_DN2758_c0_g2_i3.p1	1.57e-172	485.0	COG1028@1|root,KOG0725@2759|Eukaryota,38FP8@33154|Opisthokonta,3NVM2@4751|Fungi,3QNRJ@4890|Ascomycota,20E97@147545|Eurotiomycetes,3S7VT@5042|Eurotiales	4751|Fungi	Oxidoreductase, short-chain dehydrogenase reductase family	adh_short_C2
TRINITY_DN19225_c0_g1_i1.p1	8.6e-305	839.0	COG1252@1|root,KOG2495@2759|Eukaryota,38G4F@33154|Opisthokonta,3NW0S@4751|Fungi,3QS42@4890|Ascomycota,20CQ9@147545|Eurotiomycetes,3S3IQ@5042|Eurotiales	4751|Fungi	Pyridine nucleotide-disulphide oxidoreductase	Pyr_redox_2
TRINITY_DN3586_c0_g1_i4.p3	3.57e-131	392.0	COG2072@1|root,KOG1399@2759|Eukaryota,38D7U@33154|Opisthokonta,3NUI0@4751|Fungi,3QQWG@4890|Ascomycota,210ZU@147548|Leotiomycetes	4751|Fungi	Pyridine nucleotide-disulphide oxidoreductase	FMO-like
TRINITY_DN19170_c0_g1_i1.p1	1.58e-189	532.0	COG0604@1|root,KOG1198@2759|Eukaryota,39UN2@33154|Opisthokonta,3NYHK@4751|Fungi,3QJGP@4890|Ascomycota,20EVP@147545|Eurotiomycetes,3S4ZY@5042|Eurotiales	4751|Fungi	Quinone oxidoreductase	ADH_N

TRINITY_DN2532_c0_g1_i2.p1	1.64e-195	547.0	COG0604@1|root,KOG1198@2759|Eukaryota,39UN2@33154|Opisthokonta,3NYHK@4751|Fungi,3QJGP@4890|Ascomycota,20EVP@147545|Eurotiomycetes	4751|Fungi	Quinone oxidoreductase	ADH_N,ADH_zinc_N

Table S8: Results of the differentially expressed genes analysis of the fungus *Aspergillus puulaauensis* (EXF-7678) grown on synthetic material Regalrez 1094, obtained using the EggNOG online database. Genes for esterases and oxidases with increased expression levels are shown. The orange color represents the group of esterases, blue oxidase and yellow oxidoreductase. Genes whose predicted proteins have secretory (Sec) pathway targeting signal sequences are in bold.

query	evalue	score	eggNOG_OGs	max_annot_lvl	Description	PFAMs
**TRINITY_DN5146_c0_g1_i3.p2**	3e-186	521.0	COG3509@1|root,2QTDU@2759|Eukaryota,39SMP@33154|Opisthokonta,3NZTT@4751|Fungi,3QMXZ@4890|Ascomycota,20K1Z@147545|Eurotiomycetes,3S35W@5042|Eurotiales	4751|Fungi	Acetylxylan esterase involved in the hydrolysis of xylan. Degrades	CBM_1,Esterase_phd
TRINITY_DN6124_c0_g1_i1.p2	1.38e-109	326.0	COG2819@1|root,2SII4@2759|Eukaryota,3AKDR@33154|Opisthokonta,3PDMH@4751|Fungi,3RMNN@4890|Ascomycota,20U4J@147545|Eurotiomycetes	4751|Fungi	Putative esterase	Esterase
TRINITY_DN1702_c0_g1_i1.p2	4.88e-33	127.0	29DZU@1|root,2RM4B@2759|Eukaryota,39X15@33154|Opisthokonta,3NVC7@4751|Fungi,3QKJG@4890|Ascomycota,20DK8@147545|Eurotiomycetes,3SA4A@5042|Eurotiales	4751|Fungi	Common central domain of tyrosinase	Tyrosinase
TRINITY_DN1702_c0_g1_i1.p1	2.38e-61	201.0	29DZU@1|root,2RM4B@2759|Eukaryota,39X15@33154|Opisthokonta,3NVC7@4751|Fungi,3QKJG@4890|Ascomycota,20DK8@147545|Eurotiomycetes,3SA4A@5042|Eurotiales	4751|Fungi	Common central domain of tyrosinase	Tyrosinase
TRINITY_DN11_c0_g2_i1.p1	7.51e-130	382.0	COG2130@1|root,KOG1196@2759|Eukaryota,39AU4@33154|Opisthokonta,3NWMF@4751|Fungi,3QPK5@4890|Ascomycota,20DHF@147545|Eurotiomycetes	4751|Fungi	N-terminal domain of oxidoreductase	ADH_N_2,ADH_zinc_N

Table S9: Results of the differentially expressed genes analysis of the fungus *Aspergillus puulaauensis* (EXF-7678) grown on synthetic material Regalrez 1094, obtained using the EggNOG online database. Genes for esterases and oxidases with reduced expression levels are shown. The orange color represents the group of esterases, blue oxidase and yellow oxidoreductase. Genes whose predicted proteins have secretory (Sec) pathway targeting signal sequences are in bold.

query	evalue	score	eggNOG_OGs	max_annot_lvl	Description	PFAMs
TRINITY_DN1374_c0_g1_i1.p4	1.86e-68	218.0	28PJD@1|root,2QW7I@2759|Eukaryota,39TJA@33154|Opisthokonta,3NWYV@4751|Fungi,3QM7J@4890|Ascomycota,20PGF@147545|Eurotiomycetes,3S7FW@5042|Eurotiales	4751|Fungi	Pectinolytic enzyme	-
**TRINITY_DN1374_c0_g1_i1.p3**	5.18e-63	205.0	28PJD@1|root,2QW7I@2759|Eukaryota,39TJA@33154|Opisthokonta,3NWYV@4751|Fungi,3QM7J@4890|Ascomycota,20PGF@147545|Eurotiomycetes,3S7FW@5042|Eurotiales	4751|Fungi	Pectinolytic enzyme	-
**TRINITY_DN27500_c0_g1_i1.p1**	4.69e-267	734.0	2CNH4@1|root,2QW99@2759|Eukaryota,392S2@33154|Opisthokonta,3NY4Y@4751|Fungi,3QMJP@4890|Ascomycota,20BII@147545|Eurotiomycetes,3S5PD@5042|Eurotiales	4751|Fungi	Glycerophosphoryl diester phosphodiesterase family protein	GDPD
**TRINITY_DN31518_c0_g1_i1.p5**	1.21e-109	328.0	COG3509@1|root,2QTDU@2759|Eukaryota,39SMP@33154|Opisthokonta,3NZTT@4751|Fungi,3QMXZ@4890|Ascomycota,20K1Z@147545|Eurotiomycetes,3S35W@5042|Eurotiales	4751|Fungi	Acetylxylan esterase involved in the hydrolysis of xylan.	CBM_1,Esterase_phd
TRINITY_DN1756_c0_g1_i2.p2	6.73e-27	114.0	COG2132@1|root,COG2132@2|Bacteria,3Y65V@57723|Acidobacteria,2JMCT@204432|Acidobacteriia	204432|Acidobacteriia	PFAM multicopper oxidase type 2	Cu-oxidase,Cu-oxidase_2,Cu-oxidase_3
**TRINITY_DN1756_c0_g1_i2.p1**	3.22e-22	102.0	COG2132@1|root,arCOG03914@2157|Archaea,2XUY3@28890|Euryarchaeota,23U0K@183963|Halobacteria	183963|Halobacteria	Multicopper	Cu-oxidase_2,Cu-oxidase_3
**TRINITY_DN2239_c0_g1_i6.p1**	6.9e-273	761.0	COG1231@1|root,KOG0029@2759|Eukaryota,38F9V@33154|Opisthokonta,3NTXA@4751|Fungi,3QKSW@4890|Ascomycota,20EFU@147545|Eurotiomycetes,3S854@5042|Eurotiales	4751|Fungi	Polyamine oxidase	Amino_oxidase
TRINITY_DN51_c0_g4_i1.p2	1.47e-154	458.0	COG2124@1|root,KOG0156@2759|Eukaryota,38BSU@33154|Opisthokonta,3NUFV@4751|Fungi,3QSVE@4890|Ascomycota,20FN2@147545|Eurotiomycetes,3SCJJ@5042|Eurotiales	4751|Fungi	Catalyzes the conversion of tryprostatin A to fumitremorgin C	p450
TRINITY_DN2532_c0_g1_i2.p1	1.64e-195	547.0	COG0604@1|root,KOG1198@2759|Eukaryota,39UN2@33154|Opisthokonta,3NYHK@4751|Fungi,3QJGP@4890|Ascomycota,20EVP@147545|Eurotiomycetes	4751|Fungi	Quinone oxidoreductase	ADH_N,ADH_zinc_N
TRINITY_DN2758_c0_g2_i3.p1	1.57e-172	485.0	COG1028@1|root,KOG0725@2759|Eukaryota,38FP8@33154|Opisthokonta,3NVM2@4751|Fungi,3QNRJ@4890|Ascomycota,20E97@147545|Eurotiomycetes,3S7VT@5042|Eurotiales	4751|Fungi	Oxidoreductase, short-chain dehydrogenase reductase family	adh_short_C2
TRINITY_DN51_c0_g4_i1.p3	4.25e-44	156.0	COG3491@1|root,COG3491@2|Bacteria,1MUNT@1224|Proteobacteria,1RSQ2@1236|Gammaproteobacteria,1Z1SJ@136846|Pseudomonas stutzeri group	1236|Gammaproteobacteria	Belongs to the iron ascorbate-dependent oxidoreductase family	2OG-FeII_Oxy,DIOX_N

TRINITY_DN5247_c0_g1_i1.p1	0.0	1117.0	COG2303@1|root,KOG1238@2759|Eukaryota,38E77@33154|Opisthokonta,3NVXQ@4751|Fungi,3QRKP@4890|Ascomycota	4751|Fungi	Belongs to the GMC oxidoreductase family	CDH-cyt,GMC_oxred_C,GMC_oxred_N

2 Supplementary Figures

Figure S1: Esterase activities of selected fungi, expressed in µmol of 4-nitrophenyl (4-NP) substrate/mg of fungal supernatant. Data are represented as means ± standard deviation (SD) (n = 3).

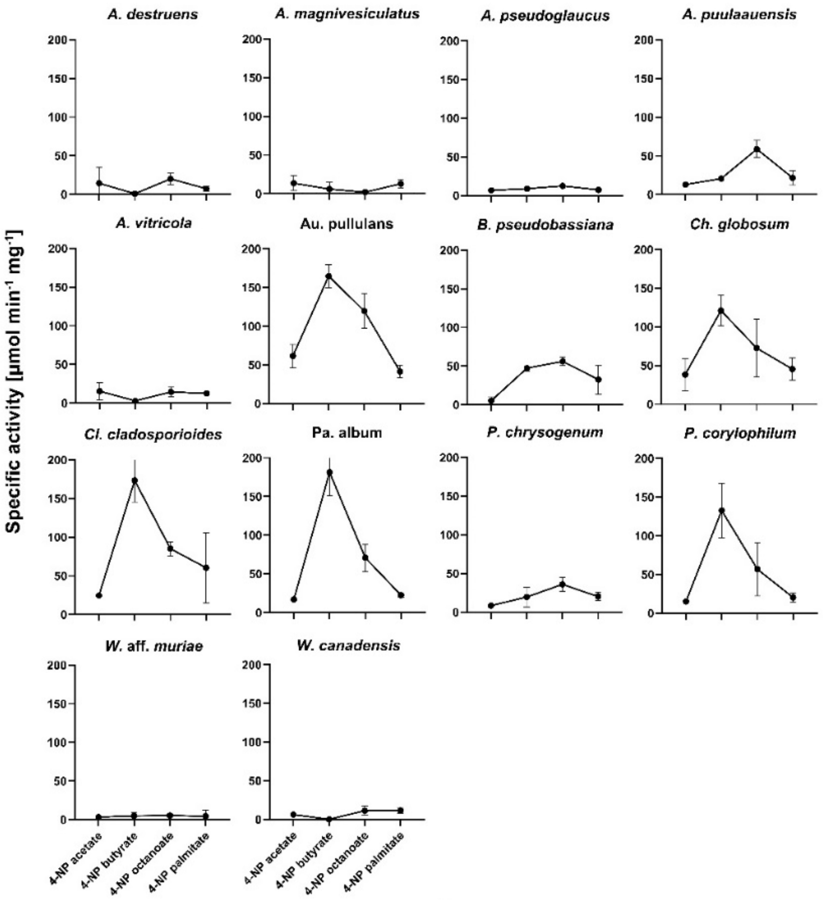

Supplement: Supplementary file 1 [file jof-11-00568-s001.zip › jof-3754665-supplementary.pdf]
